# Zinc transport from the endoplasmic reticulum to the cytoplasm via Zip7 is necessary for barrier dysfunction mediated by inflammatory signaling in RPE cells

**DOI:** 10.1371/journal.pone.0271656

**Published:** 2022-07-28

**Authors:** YongYao Xu, Michael Twarog, Ning Li, Angela Banks, Josh Schustak, Yi Bao, Qian Huang, Quintus G. Medley

**Affiliations:** Department of Ophthalmology, Novartis Institutes of Biomedical Research, Cambridge, MA, United States of America; University of Illinois at Chicago, UNITED STATES

## Abstract

Inflammatory signaling induces barrier dysfunction in retinal-pigmented epithelium (RPE) cells and plays a role in the pathology of age-related macular degeneration (AMD). We studied the role of Zn flux from the endoplasmic reticulum (ER) to the cytoplasm via Zip7 during inflammatory signaling in RPE cells. In ARPE-19 cells, Zip7 inhibition reduced impedance loss, FITC-dextran permeability and cytokine induction caused by challenge with IL-1β/TNF-α. Zip7 inhibition in iPS-derived RPE cells challenged with TNF- α reduced barrier loss in TER assays. In ARPE-19 cells, a Zn ionophore restored cytokine induction and barrier loss in cells challenged with IL-1 β /TNF- α despite Zip7 inhibition. A cell permeable Zn chelator demonstrated that Zn is essential for IL-1 β /TNF- α signaling. ER stress caused by Zip7 inhibition in ARPE-19 cells was found to partially contribute to reducing barrier dysfunction caused by IL-1 β /TNF- α. Overall, it was shown that Zn flux through Zip7 from the ER to the cytoplasm plays a critical role in driving barrier dysfunction caused by inflammatory cytokines in RPE cells.

## Introduction

The barrier function of retinal endothelial cells and retinal pigmented epithelial (RPE) cells maintain retinal homeostasis and exchange material into and out of the retina, protect the inner and outer retina from pathogens derived from the circulation and regulate inflammatory cell access to the retina [1]. Dysfunction of the protective barrier activity of these cells are hallmarks of diabetic macular edema (DME) and AMD [2–4].

RPE cells form a barrier in the outer retina as well as mediating exchange of metabolites between the choroid and retina. During AMD, the RPE cell layer becomes irregular as a result of disruption of the junctions between cells and the cells can superimpose upon one another and open gaps within the monolayer. The disruption of the RPE layer compromises the ability of the RPE to support the photoreceptors [5, 6]. Late stage neovascular AMD occurs when vessels grow from the choroidal tissue through the RPE layer and into the retina, causing blindness. During AMD, microglia migrate from the inner retina down to the RPE layer [7] providing a source of inflammatory cytokines (such as IL-1β, IL-6, IL-8 and TNF-α (reviewed in [8]) that can act directly on the RPE layer. It has therefore been postulated that inflammation is a contributing factor in the pathogenesis of AMD [9]. The treatment of diabetic macular edema (DME) with corticosteroids also argues for the importance of inflammation to its disease pathology [10, 11]. TNF- α and IL-1 β were shown to alter the permeability of endothelial cell monolayers and reduce levels of tight junction proteins [12].

Cytosolic zinc (Zn) is an important element that is incorporated into many proteins structurally and has been shown to act as an intracellular second messenger [13–15]. The role of Zn in cell signaling has been shown to be both activating and inhibitory. In T cells, Zn increased IL-2 induced by IL-1β and Zn flux mediated by Zip8 increased T cell signaling including IFN-γ production [16, 17]. Zn is also required for TLR4 signaling in leukocytes [18, 19] and for Fc receptor signaling in mast cells [20]. The role of Zn in RPE is beginning to be elucidated and Zn depletion was shown to lead to disruption of a number of pathways and cell death reviewed in [13].

Zip7 is a member of a Zn transporter family of proteins that convey Zn into the cytoplasm of cells from intracellular compartments or from the extracellular environment. Zip7 is the sole Zn transporter that resides in the ER membrane and pumps Zn from the ER to the cytoplasm [21]. Zip7 activity is responsive to signaling pathways and is activated by phosphorylation by CK2 [22]. Previous work identified a Zip7 small molecule inhibitor NVS-ZP7-1 using a phenotypic screen to identify inhibitors of Notch signaling [23].

In this paper we establish a previously unappreciated link between Zn and inflammatory signaling causing barrier dysfunction in the retina. We show for the first time in RPE cells a role for Zn in the signaling pathways downstream of IL-1 β /TNF- α in cytokine induction and in mediating barrier dysfunction. Additionally, we show a role for Zn in the TNF- α signaling pathway in induced pleuripotent stem cell-derived (iPS) RPE cells. Moreover, the use of a small molecule inhibitor revealed a key role for Zip7 in regulation of RPE Zn levels. Modulating Zn flux, such as by Zip7 inhibition, may represent a novel therapeutic approach to treat ocular diseases such as AMD and DME.

## Materials and methods

### Compounds, recombinant proteins and antibodies

NVS-ZP7-4 is an inhibitor of Zip7 that was derived from a compound (NVS-ZP7-1) that was obtained in a phenotypic screen of inhibitors of Notch signaling as previously described [23]. NVS-ZP7-6 is an inactive analog of NVS-ZP7-4. NVS-ZP7-1, NVS-ZP7-4 and NVS-ZP7-6 were synthesized internally at Novartis. Tauroursodeoxycholic acid (TUDCA) (cat# T0266), salubrinal (cat# 324895), dibenzazepine (cat# 209984-56-S) and compound E (cat# 209986-17-4) were purchased from Sigma. All recombinant proteins were purchased from R&D Systems, Minneapolis, MN, unless otherwise noticed. TPEN (N,N,N′,N′-Tetrakis(2-pyridylmethyl)ethylenediamine) was purchased from Sigma (cat# P4413). DTPA (diethylenetriaminepentaacetic acid) was purchased from Sigma (cat# D1133). The following antibodies were purchased from Cell Signaling Technologies and used at 1:1000 dilution: BiP (C50B12) rabbit mAb #3177, IRE1 α (14C10) rabbit mAb #3294, CHOP (L63F7) mouse mAb #2985, calreticulin (D3E6) rabbit mAb #12238, PERK (C33E10) rabbit mAb #3192, XBP-1s (D2C1F) rabbit mAb #12782, ZIP7/SLC39A& (D103A) rabbit mAb # 33176. The following antibodies were purchased from Cell Technologies and used at 1:2000 dilution: GAPDH (14C10) rabbit mAb #2118, β -actin (8H10D10) mouse mAb #3700, goat anti-rabbit HRP-linked IgG #7074, horse anti-mouse HRP-linked IgG #7076.

### Cell culture

ARPE-19 cells were purchased from ATCC (cat# CRL-2302) and cells were grown in DMEM F12 media with 10% FBS (Thermo Fisher). iPS-RPE were purchased from FUJIFILM Cellular Dynamics, Inc. cat# R1113 and grown in RtEBM Retinal Epithelial Cell Basal Medium from Lonza (cat# 00195406) with 2% FBS. Both cells were plated at confluence for 3 weeks prior to use in experiments as per the supplier’s protocols.

### Cell impedance assay

Impedance experiments were carried out using the xCelligence Real Time Cell Analysis (RTCA) platform (Acea Biosciences, California). Human retinal microvascular endothelial cells (HREC) (Neuromics, Edina, MN) or ARPE-19 (ATCC, Gaithersburg, Maryland) were seeded in each well (E-plate 96, ACEA Biosciences) and cultured until confluent. Cells were then cultured in media containing NVS-ZP7-1 or NVS-ZP7-4 with various ligands. Assays using HREC used TNF- α, 5ng/ml; IL-1 β, 5ng/ml; AGE-BSA (250μg/ml, BioVision, Milpitas, CA or VEGF(50ng/ml) whereas ARPE-19 used IL-1β (5 ng/mL)/TNF- α 10 ng/mL). Typically cells were incubated with compound 30 minutes prior to inflammatory challenge and impedance measured for 18h after cytokine addition. Cell viability in the assay was measured using CellTiter glo Luminescent Cell Viability Assay (Promega, Fitchburg, WI). The impedance value of each well was automatically monitored by the xCELLigence system and expressed as a CI (cell index) value. Data for cell impedance were normalized to the value at the time of ligand addition. Normalized CI is calculated using the software provided by the vendor, end time point reading was used for calculation of “% of increase”, which is equal to (CI of compound with ligand-CI of media)/(CI of ligand alone-CI of media)*100%.

### RNA isolation and real time-PCR

ARPE-19 cells were seeded at near confluence (40k cell per well of a 96-well plate) and cultured for an additional 3–4 weeks. Confluent wells were harvested (approximately 50K cells/well) and were lysed and mRNA prepared using using TurboCapture 96 mRNA kit (Qiagen, Germantown, MD) according to the manufacturer’s protocol. mRNA was used directly in cDNA synthesis by High-capacity cDNA reverse Transcription kit (Thermo Fisher, Bedford, MA) according to the manufacturer’s protocol. Real-Time PCR reaction was carried out on a ViiA 7 machine (Thermo Fisher) with TaqMan Gene Expression Assays using TaqMan Fast Advanced Master mix from Thermo Fisher. All data normalized to β-Actin expression. The delta–delta Ct method was used as described by Thermo Fisher to determine the relative levels of mRNA expression between experimental samples and controls.

XBP1 slicing primer design and its detection: as described [24, 25]. Primers were designed to span the 26 base pair intron that is removed by the splice factor IRE1 to produce spliced XBP1 mRNA (forward 5′TGCTGAGTCCGCAGCAGGTG3′ and reverse 5′GCTGGCAGGCTCTGGGGAAG3′).

### siRNA knockdown of ZIP7 in ARPE-19

ARPE-19 cells were cultured for 3 weeks in 6-well plate (Corning) or E-plate (ACEA Biosciences). For siRNA knockdown, media was removed and replaced with siRNA mixture (10nM final) using Dharmafect 4 (Dharmacon). For Zip7 cDNA overexpression, 0.5-2ug ZipF7 plasmid DNA (ORF in pcDNA-DEST40 vector, Thermo Fisher) was transfected using 200ul of Opti-MEM® I Reduced-Serum Medium (Thermo Fisher) mixed with 1.5-6uL FugeneHD (Promega, Fitchburg, WI), incubated 10 min. and was added dropwise to well. The cells are then cultured 72hr at 37°C before either harvesting for western blot analysis as described or used in cell impedance assays as described.

### In vitro permeability assay

To determine paracellular permeability, ARPE-19 cell were seeded in Transwell filters (6.5 mm, 0.4 μm pore size, Corning Inc, Corning, NY) and grown to confluence. ARPE-19 cell were serum-starved overnight with 1% FBS, pre-incubated with 10μM NVS-ZP7-4 or vehicle for 30 min, followed by stimulation with IL-1 β (10ng/ml) and TNF- α (10ng/ml) for 72hr, before adding 25 mg/ml FITC-dextran (40 kD, Sigma-Aldrich, St. Louis, MO) to the top chamber. The cells were incubated at 37°C and 5% CO_2_ for an additional 2 hr and monolayer permeability to FITC-dextran determined by measuring fluorescence in the lower chamber in an EnVision Multimode Plate Reader (PerkinElmer, Waltham, MA), subtracting basal monolayer permeability.

### Western blot

For western blot analysis of each sample, 2X10^6^ cells were lysed in 1× Cell Lysis buffer (Cell Signaling Technologies Danvers, MA) containing 20 mM Tris–HCl pH 7.5, 150 mM NaCl, 1 mM EDTA, 1 mM EGTA, 1% Triton, 2.5 mM sodium pyrophosphate, 1 mM beta-glycerophosphate, 1 mM Na_3_VO_4_, 1 μg/ml leupeptin, supplemented with HALT protease and phosphatase inhibitor cocktail (Thermo Fisher, Bedford, MA). Total protein (30 μg) was electrophoresed per lane on a 4–20% Bis–Tris SDS–PAGE gel (Bio-Rad, Portland, ME). Proteins were transferred to nitrocellulose membranes using Trans-Blot Turbo Transfer System (Bio-Rad, Portland, ME). Membranes were incubated for 1 h at room temperature in 5% milk dissolved in 1× TBST (Boston Bioproducts, Inc, Ashland, MA). Membranes were incubated with primary antibodies according to supplier instructions. After incubation, membranes were washed with 1× TBST buffer for 3 × 10 min at room temperature, and probed with a 1:2000 dilution of either goat anti-rabbit IgG or horse anti-mouse IgG conjugated to horseradish peroxidase (Cell Signaling Technology) in blocking buffer for 1 h at room temperature. Following 3 × 10 min wash in 1× TBST at room temperature the blots were developed using SuperSignal™ West Pico PLUS Chemiluminescent Substrate (Thermo Fisher). The antibodies used were purchased from Cell Signaling Technologies and used at the recommended dilution: BiP (C50B12) rabbit mAb #3177 1:1000, IRE1α (14C10) rabbit mAb #3294 1:1000, CHOP (L63F7) mouse mAb #2895 1:1000, Calreticulin (D3E6) XP® rabbit mAb #12238 1:1000, PERK (C33E10) rabbit mAb #3192 1:1000, XBP-1s (D2C1F) rabbit mAb #12782 1:1000, ZIP7/SLC39A7 (D1O3A), rabbit mAb #33176 1:1000, GAPDH (14C10) rabbit mAb #2118 1:2000, ß-Actin (8H10D10) mouse mAb #3700 1:2000, goat anti-rabbit IgG #7074 1:2000, horse anti-mouse IgG #7076 1:2000. Raw images for the Western blots are shown in S1 Raw images.

### FACS analysis to assess IL1R1 on ARPE-19 cell surface

Confluent ARPE-19 cells were cultured for 3 weeks, serum starved (1% FBS) overnight, then treated with IL-1 β (5ng/ml) alone or IL-1 β plus NVS-ZP7-4 (10 μM) for 2hr or 24hr before harvesting for FACS analysis. Cells were harvested and diluted to 1X10^6^ cells per ml in FACS buffer (PBS; 0.1% BSA), total of 2X10^5^ cells per well were added to each well of a 96-well plate and centrifuged at 400Xg for 3 min at 4°C before removing the supernatant. 50 μl of human IL-1RI FITC-conjugated Antibody (R&D) or goat isotype control (R&D) was added to the cell pellets and incubated for 30min at 4°C. The cells were washed and pelleted twice with 200 μl FACS buffer then resuspended in 200 μl FACS buffer and fluorescence values were measured with a BD FACSCanto II cytometer (BD Biosciences, Franklin Lakes, NJ). The amount of cell surface-bound anti-IL1R1-FITC was assessed by measuring the mean channel fluorescence of 10K collected cell events. Cells were initially gated using forward and side scatter properties to isolate single cells. Data for each sample were then plotted on a histogram with FITC-positive cells having values above the negative isotype control sample (not shown). Data were analyzed using FlowJo7.5.5. Mean fluorescence intensity (MFI) or percentage of positive cell were plotted in the graph to compare level of expression of IL-1R1.

### ELISA for IL-6

IL-6 was used as a marker for activity downstream of IL-1β or TNF- α due to the high levels of induction. It was measured using human IL-6 DuoSet ELISA kit (R&D, Minneapolis, MN), with a modified protocol. All incubations were at room temperature. 96-well white polystyrene microplate (Corning) was coated with capture antibody at 4μg/ml in PBS overnight. Plates were then washed with buffer (PBS with 0.05% Tween 20, Sigma) three times, and then blocked with PBS containing 0.2% I-block (Thermo Fisher Scientific) with 0.05% Tween 20 for 1hr. After washing, appropriately diluted cell media was added to the plate along with human recombinant IL-6 as standard and incubated for 1hr with gentle shaking. After washing, detection antibody biotinylated goat anti-human IL-6 was added and incubated for 1hr. Streptavidin-HRP (1:40) was added to each well after washing and incubated for 30min. After washing, SuperSigna ELISA Femto substrate (Thermo Fisher) was added and the chemiluminescent signal was read in EnVision Multimode Plate Reader (PerkinElmer). The IL-6 concentration was calculated based on the standard curve of human IL-6 recombinant protein used in the assay and cell viability was determined by CellTiter-Glo (CTG) Luminescent Cell Viability Assay (Promega, Fitchburg, WI), according to the manufacturer’s guide.

### Caspase 3/7 assay

To assess if NVS-ZP7-4 was stimulating the activity of caspase 3/7, an indicator of apoptosis, the Caspase-Glo 3/7 assay system (Promega, Fitchburg, WI) was used. Included in this assay were control compounds including Thapsigargin (Sigma), which is known to induce apoptosis, along with negative controls that included DMSO and NVS-ZP7-6 (inactive version of NVS-ZP7-4). The ARPE-19 was cultured, serum starved in 1% FBS overnight, then treated with IL-1 β (5ng/ml) alone or IL-1 β plus NVS-ZP7-4 (2 μM or 10 μM) for 24hr. Caspase-Glo 3/7 reagent (Promega) was prepared according to the manufacture’s protocol and added to the cells 24 h after compound addition. Each sample was incubated with the Caspase-Glo 3/7 reagent for 30 min and then luminescence (relative luminescence units) was read on the EnVision Multimode Plate Reader (PerkinElmer).

### Intracellular zinc assay

A fluorescent Zn assay was used to determine cytoplasmic levels of Zn. ARPE19 cells were cultured for 3 weeks in 96-well flat clear bottom black polystyrene microplates (Corning), Cells were washed twice with HBSS and incubated for 30 min at 37°C with HBSS containing FluoroZin3 (final 2.5 μM, Thermo Fisher), ER-Tracker Red (final 2 μM)and NucBlue™ Live ReadyProbes™ Reagent (Hoechst 33342) (both from Thermo Fisher) and washed twice with HBSS and replaced with FluoroZin3 (final at 2.5 μM, Thermo Fisher) in HBSS, with ER-Tracker Red (final at 2 μM) and NucBlue™ Live ReadyProbes™ Reagent (Hoechst 33342) (both from Thermo Fisher). Plates were washed with Hank’s Balanced Salt Solution (HBSS, Thermo Fisher) four times; RPMI 1640 Medium (no phenol red, Thermo Fisher) with ZnCl2 (5 μM) and sodium pyrithione (10 μM) (both from Sigma) was added. Plates were incubated for 2hr at 37°C. Live images were taken using IN Cell Analyzer 2200 (GE Healthcare Life Sciences). Co-staining of Zn and the endoplasmic reticulum were done by preloading ARPE-19 cells with endoplasmic reticulum (ER) specific sensor ZBR3 for 30min, wash thoroughly with HBSS, then add NVP-ZIP7-4 and zinc/pyrithione for 2 hr, before taking the live image using INCELL6500.

### ZO-1 immunostaining

ARPE-19 cells were maintained confluent for 3weeks then cultured overnight in media containing 1% FBS. Cells were treated with NVS-ZP7-4 (10 μM) or IL-1 β /TNF- α (10ng/ml) or both for 24 hr. The media was removed and cells fixed with 4% paraformaldehyde for 15min and washed twice with PBS. Cells were blocked with 5% goat serum (Abcam, Cambridge, MA) for 1 hr and incubated with ZO-1 Monoclonal Antibody (ZO1-1A12), Alexa Fluor 488 (5μg/ml, Thermo Fisher) overnight at 4°C. The supernatant was removed and F-actin was stained for using Alexa Fluor™ 594 Phalloidin (Thermo Fisher) added at 0.8 μM final and nuclei stained for using DAPI at 2.5 μg/ml final (Thermo Fisher). After incubating for 20 minutes, the plate was washed with PBS three times, before imaging with INCELL2200.

### iPS-derived RPE transepithelial resistance (TER)

iPS-derived RPE cells were generated from human pluripotent stem cells as previously described [26]. Cells were cultured in Lonza RtEGM medium (Lonza, Basel, Switzerland) at 37°C and 5% CO_2_. iPS-RPE cells were cultured on Falcon transmembrane inserts for polarization (Corning, Corning, NY; 353095), and cultured for at least 4 weeks post-seeding to form mature monolayers. Barrier function of RPE cells was assessed by monitoring TER (Ωcm^2^) every 15 minutes by means of a cellZscope 2 (NanoAnalytics GmbH, Münster, Germany).

### Statistical analysis

Statistical significance was analyzed using one-way ANOVA for independent samples. Tukey’s multiple comparison test was applied to correct for multiple comparisons. GraphPad Prism7 software was used for this analysis. P-values are indicated by asterisks: *P < 0.05; **P < 0.01; and ***P < 0.001. Results are shown as means+/-SEM. Representative experiments are shown from three independent experiments each using n = 3 replicates.

## Results

### Zip7 inhibition prevents the loss of barrier function of RPE cells caused by inflammation

**Fig 1:**
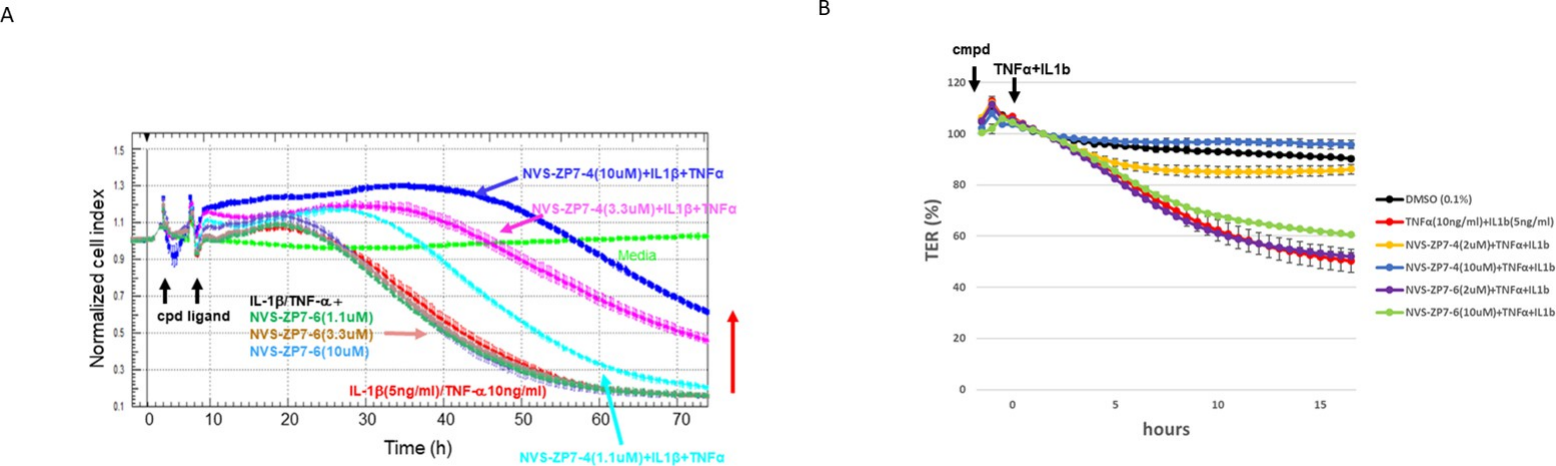
Zip7 zinc transport is involved in IL-1 β /TNF- α signaling in RPE cells. A: Impedance was measured in confluent ARPE-19 cells for 72h in media, media + IL-1 β (5 ng/mL)/TNF- α (10ng/mL), media + IL-1 β /TNF- α + NVS-ZP7-4 or media + IL-1 β /TNF- α + NVS-ZP7-6 (inactive analog of NVS-ZP7-4). B: Challenge of human iPS-derived RPE cells by IL-1β /TNF- α in the presence of NVS-ZP7-4 increased transepithelial resistance relative to cells treated with IL-1β /TNF- α alone. Inclusion of NVS-ZP7-6 during challenge with IL-1β /TNF- α resulted in impedance loss.

Zip7 is a Zn transporter expressed in the membrane of the er and transports Zn from the er into the cytoplasm. A selective Zip7 inhibitor NVS-ZP7-4 is related to a compound originally identified in a phenotypic screen for Notch inhibitors (21). To understand if Zip7 plays a role in Zn transport in RPE cells, the effect of inhibition of Zip7 on inflammatory signaling was tested using ARPE-19 in impedance assays. Under the conditions of culture in the impedance assay, challenge of ARPE-19 cells with IL-1β could affect impedance although more consistent effects were observed when cells were challenged with IL-1 β /TNF- α together (Fig 1A). When the Zip7 inhibitor NVS-ZP7-4 was present during the challenge, little change in impedance was observed (Fig 1A), indicating that inhibition of the transport Zn from the er to the cytoplasm by Zip7 reduced the ability of IL-1 β /TNF- α to cause cellular changes mediating impedance loss. Inclusion of NVS-ZP7-6, the inactive analog of NVS-ZP7-4, had no effect on the activity of IL-1 β /TNF- α suggesting the activity of NVS-ZP7-4 was specific. Treatment of ARPE-19 with NVS-ZP7-4 either alone or with IL-1 β /TNF- α did not affect cell viability (as assessed by CellTiterGlo, described in Materials and Methods) or induce apoptosis (as assessed by caspase3/7 activity described in Materials and Methods).

To ensure that the effect of NVS-ZP7-4 is relevant to RPE cells and not a phenomenon associated with the ARPE-19 cell line, we tested the effect of Zip7 inhibition in transepithelial cell resistance (TER) assays using RPE cells differentiated from iPS cells. TER assays measure resistance across confluent cells cultured on a transwell cell culture inserts. In this system iPS-derived RPE achieve much higher levels of resistance than ARPE-19 indicating that they form much more robust tight junctions associated with primary RPE cells [27]. Similar to the protective effect on barrier function seen in ARPE-19 cells, inclusion of NVS-ZP7-4 during inflammatory challenge with IL-1β /TNF- α maintained barrier function in iPS-derived RPE cells and prevented the loss of resistance in the TER assay (Fig 1B). The effect of the compound was selective for Zip7 inhibition as the inactive analog NVS-ZP7-6 did not protect from barrier function loss (Fig 1B). Challenge of iPS-RPE with TNF- α alone also reduced barrier function in TER assays and this loss was also prevented by NVS-ZP7-4 whereas the inactive analog NVS-ZP7-6 had no effect (S1 Fig). This demonstrates that Zn flux through Zip7 is necessary for tight junction dysfunction caused by inflammatory signaling in iPS-derived RPE cells in addition to ARPE-19 cells. Again, the protective effect was specific to NVS-ZP7-4 as its inactive analog NVS-ZP7-6 did not offer any protection from barrier function loss. Since the role of Zn in mediating barrier dysfunction during inflammatory signaling was similar in primary iPS-derived RPE and ARPE-19, hereafter all experiments used the ARPE-19 cell line.

**Fig 2:**
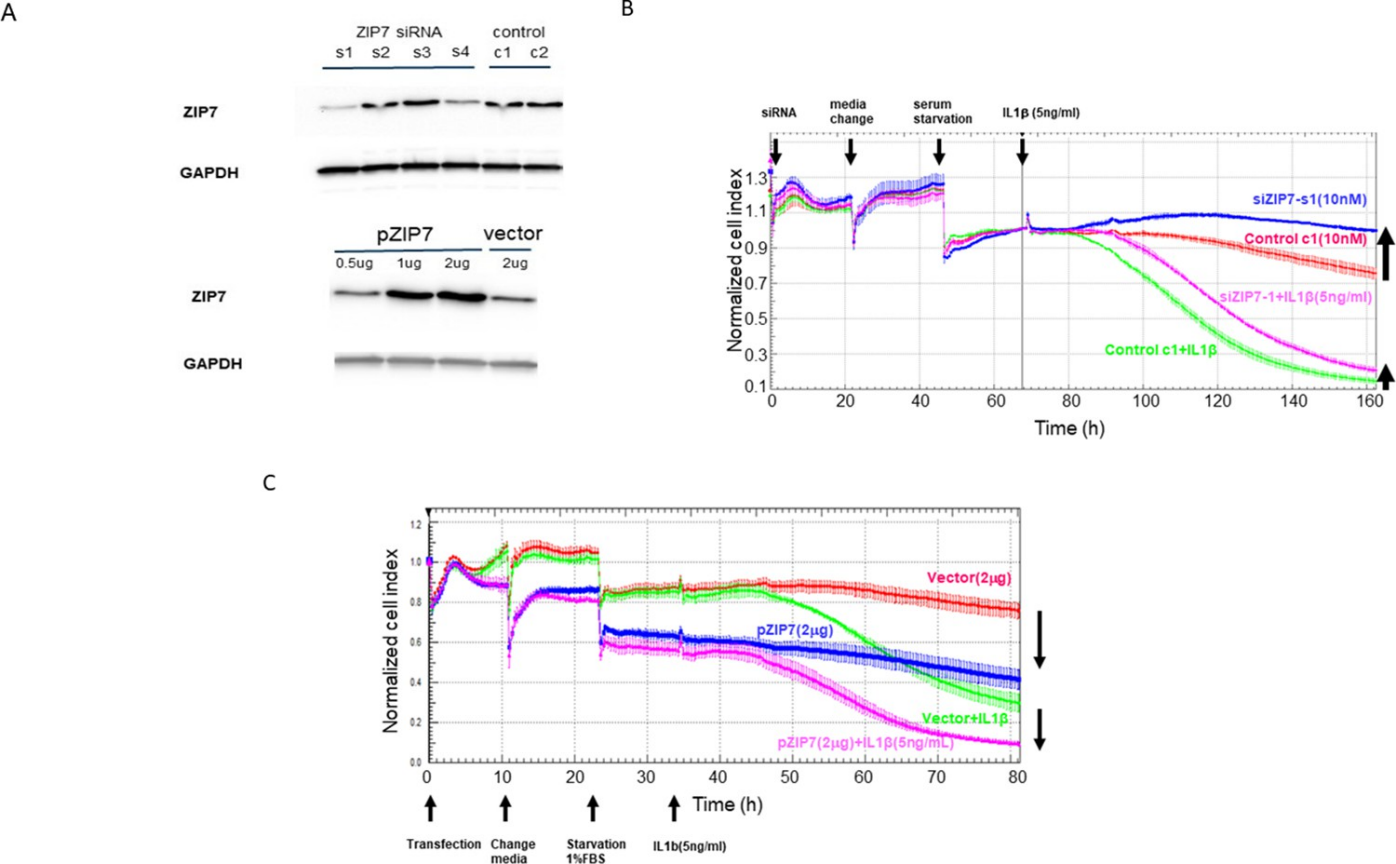
Validation of the role of Zip7 in mediating barrier dysfunction by Zip7 knockdown or overexpression. A: Cells were transfected with various siRNA oligos and after 72h lysates were made and western blot analysis performed as described in the methods section. siRNA oligo siRNA-1 and siRNA-4 both reduced levels of endogenous Zip7 whereas the expression construct caused increased levels of Zip7 protein in the cell lysates. B: Confluent ARPE-19 cells were treated with10 nM of Zip7 siRNA (s1) or control siRNA (c1) in the presence of 5 ng/mL IL-1 β. Zip7 knockdown increased impedance vs cells transfected with control siRNA. Treatment of cells with IL-1 β reduced impedance and Zip7 knockdown reduced the impedance loss indicating Zip7 contributes to barrier loss induced by IL-1 β. C: Confluent ARPE-19 cells were transfected with a Zip7 expression construct or a vector control and after 48h treated with media or 5 ng/mL of IL-1 β. Zip7 overexpression reduced impedance in resting cells and overexpression of Zip7 reduced impedance during challenge with IL-1 β more than the reduction observed after cells transfected with control vector were challenged with IL-1 β.

To validate the role of Zip7 during inflammatory cytokine signaling genetically, we knocked down or over-expressed Zip7 in cells used in impedance assays after challenge with IL-1 β /TNF- α. Western blot assays of Zip7 in cell extracts allowed us to identify 2 siRNA oligos that reduced protein levels of Zip7 (Fig 2A top). We also validated that our expression construct increased levels of Zip7 in the transfected cells (Fig 2A bottom). In agreement with the experiments using NVS-ZP7-4, we found that Zip7 knockdown reduced the effect of the cytokines on resistance in impedance assays (Fig 2B) whereas overexpression of Zip7 exacerbated the resistance loss they caused (Fig 2C). The partial effect of knockdown or overexpression of Zip7 in impedance assays is likely a result of incomplete transduction of all the confluent cells.

**Fig 3:**
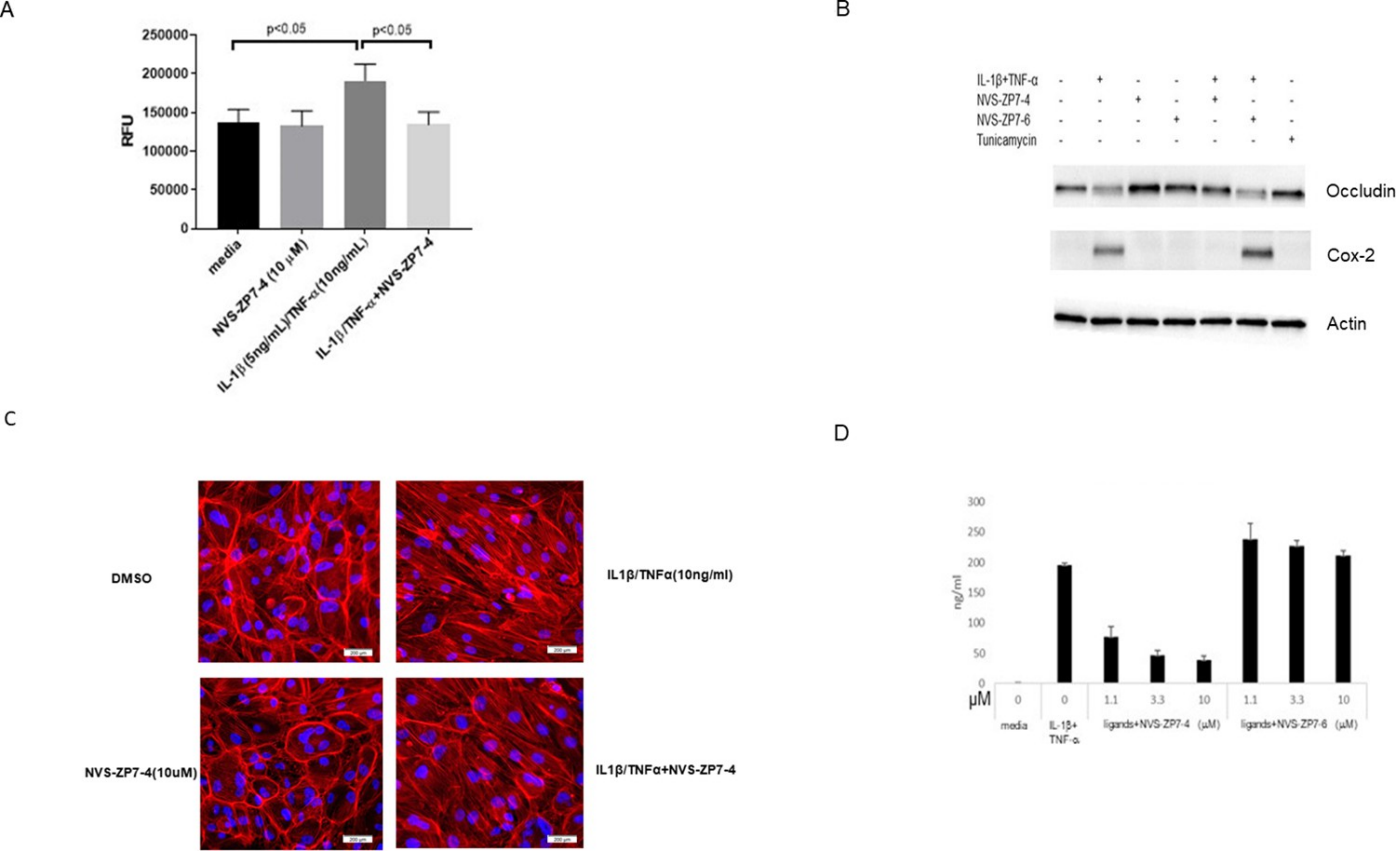
Further characterization of the effects of Zip7 inhibition on cellular barrier function: A: Confluent cells in a Transwell were treated with 5 ng/mL IL-1 β and 10 ng/mL TNF α and 10 μM NVS-ZP7-4 for 48h and 40 kDa FITC-dextran was added to the upper chamber and fluorescence was measured in the bottom chamber after 2h. Challenge of cells with cytokine increased permeability of the monolayer to FITC-dextran that was blocked by NVS-ZP7-4. B: Cells were treated with IL-1 β /TNF- α in the presence of Zip7 inhibitor NVS-ZP7-4 and inactive analog NVS-ZP7-6. After 48h, cell were harvested and a lysate was subjected to SDS-polyacryamide gel electrophoresis and western blot analysis. IL-1 β /TNF- α induced Cox-2 and reduced occludin. Inclusion of active NVS-ZP7-4 completely blocked these effects whereas NVS-ZP7-6 had no effect. C: F-actin was stained with phalloidin.IL-1 β /TNF- α challenge alone caused stress fiber formation whereas NVS-ZP7-4 treatment alone increased cortical F-actin staining. Stress fiber formation caused by IL-1 β /TNF- α was reduced by NVS-ZP7-4. D: IL-6 induction was inhibited by NVS-ZP7-4 in a dose-dependent manner whereas NVS-ZP7-6 had no effect.

To further characterize the activity of NVS-ZP7-4, we tested its effect on the permeability of a monolayer of cells challenged with IL-1 β /TNF- α to FITC-dextran. Treatment of cells with IL-1 β /TNF- α led to a statistically significant increase of FITC-dextran across the monolayer relative to untreated cells. Addition of NVS-ZP7-4 during the IL-1 β /TNF- α challenge prevented this increase and reduced the FITC-dextran transit across the monolayer to levels seen in unchallenged cells (Fig 3A). We next measured the protein and mRNA levels of proteins involved in tight junctions, and found that treatment of cells with NVS-ZP7-4 prevented a reductions of several tight junction proteins caused by IL-1 β /TNF- α challenge. Inclusion of NVS-ZP7-4 resulted in maintenance of occludin protein levels at levels seen in resting cells and a 5-fold increase in occlusion mRNA levels vs IL-1 β /TNF- α challenge alone. mRNA levels of claudin-1 were increased by 7-fold and ZO-1 by 2–3 fold when NVS-ZP7-4 was present during IL-1 β /TNF- α challenge vs IL-1 β /TNF- α challenge alone (S2 Fig). Finally, the distribution of F-actin in ARPE-19 cells changed during IL-1 β /TNF- α challenge with less cortical F-actin and more actin filament stress fibers throughout the cytoplasm. When NVS-ZP7-4 was present during challenge, an increase of stress fibers was not observed and instead strong cortical F-actin staining was maintained, similar to its localization in resting cells (Fig 3C).

Besides inhibiting barrier dysfunction induced by IL-1 β /TNF- α, NVS-ZP7-4 also inhibited IL-6 production whereas NVS-ZP7-6, an inactive analog of NVS-ZP7-4, had no effect (Fig 3D). Additionally, the induction of Cox-2 caused by IL-1 β /TNF- α was also completely blocked by NVS-ZP7-4 whereas NVS-ZP7-6 had no effect (Fig 3E). These results indicate that functioning Zip7 is necessary for other downstream effects of IL-1 β /TNF- α signaling in addition to barrier dysfunction.

### The inhibition of inflammation-driven barrier dysfunction by NVS-ZP7-4 does not involve Notch signaling nor IL-1R1 trafficking

Since NVS-ZP7-4 was originally identified through a phenotypic screen to identify inhibitors of Notch signaling, we tested whether Notch signaling was involved in mediating effects of IL-1 β /TNF- α on the barrier function of cells using γ-secretase inhibitors. The inhibitor compound E only marginally reduced barrier function loss induced by IL-1β /TNF- α in impedance assays (S3A Fig) and the inhibitor dibenzazepine (DBZ) had no effect (S3B Fig). These results indicate that the effect of Zip7 inhibition on the signaling of IL-1 β /TNF- α is likely not a result of the inhibition of Notch signaling. Previously, the mechanism of action of NVS-ZP7-4 to inhibit Notch signaling was found to be the impairment of Notch receptor trafficking. In flow cytometry assays using unpermeabilized cells, NVS-ZP7-4 was found to have no effect on levels of IL-1R1 on the cell surface during IL-1β treatment (S4 Fig). Therefore, while NVS-ZP7-4 altered the trafficking of Notch receptors to the cell surface, its inhibition of IL-1 β signaling did not appear to involve inhibition of trafficking of IL-1R1.

### Role of cytoplasmic Zn in mediating barrier dysfunction

**Fig 4:**
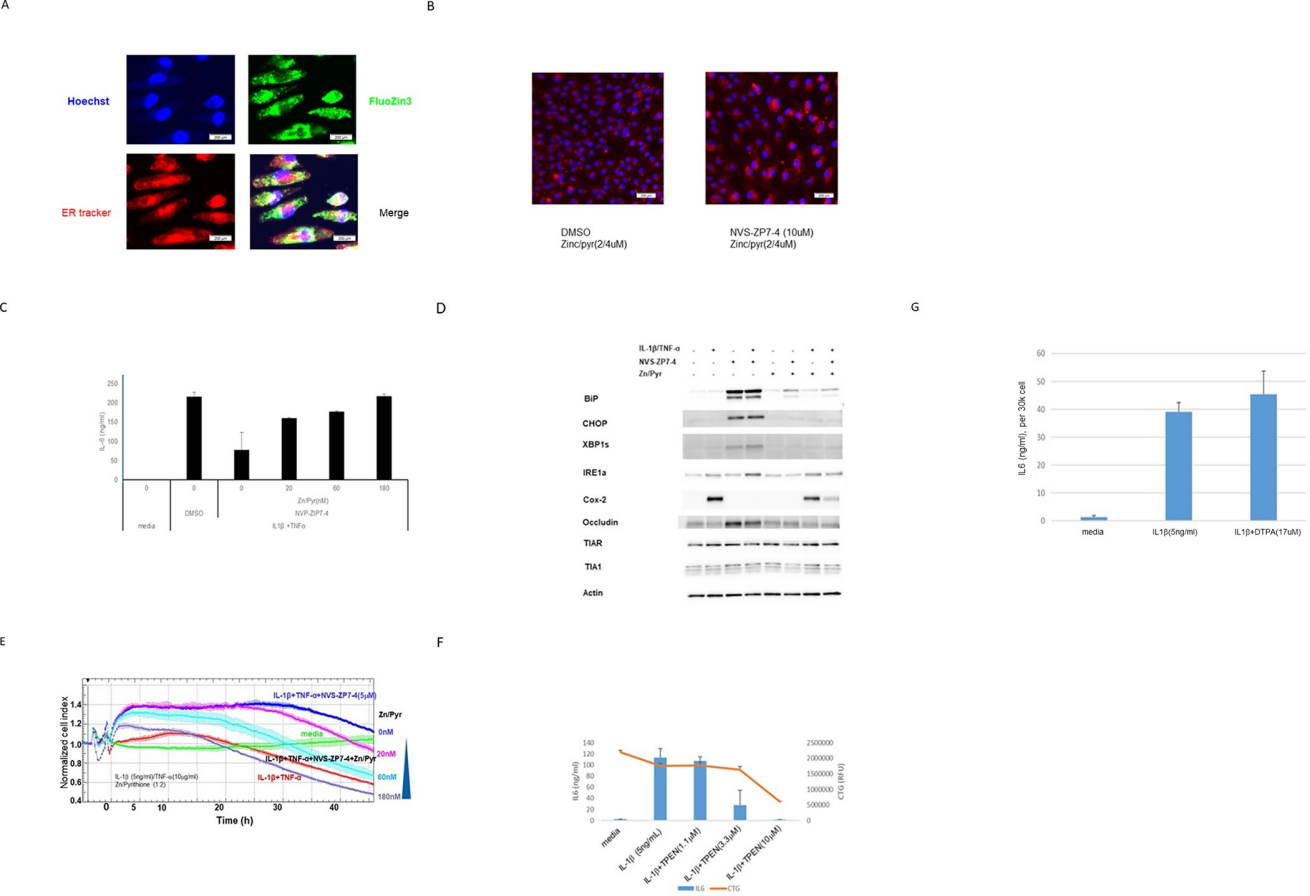
The role of Zn in signaling of IL-1 β /TNF- α or IL-1 β alone was confirmed using Zn ionophores or Zn chelators. A: ARPE-19 cells were treated with fluozin3 and ER tracker for 30min, then ZnCl2 (5 μM) and sodium pyrithione (10 μM) was added and cells were cultured for two more hours. Images were visualized with IN Cell Analyzer 2200 as described in the Methods section to visualize Zn levels in cells. Zn/Pyr increased Zn levels in the cell cytoplasm (green) and also in the ER (yellow). B: Cotreatment of ARPE-19 cells with Zn/Pyr and NVS-ZP7-4 increased Zn levels in the er as visualized with ZBR3. C: Cells were challenged with IL-1 β /TNF- α overnight in the presence of NVS-ZP7-4 and increasing concentrations of Zn/Pyr and IL-6 levels in the culture media were determined. Increasing Zn/Pyr restored IL-6 production induced by IL-1 β /TNF- α despite Zip7 inhibition with NVS-ZP7-4. D: Cells were treated with combinations of IL-1 β /TNF- α (10 ng/mL / 20 ng/mL) and western blotting analysis performed. Treatment of cells with 10 ng/mL IL-1 β and 20 ng/mL TNF- α induced Cox-2. Treatment of cells with NVS-ZP7-4 alone induced BiP, CHOP and sXBP-1 as well as occludin. Inclusion of NVS-ZP7-4 during cytokine treatment resulted in inhibition of Cox-2 induction and occludin and the er stress markers BiP, CHOP and sXBP-1. Cytokine treatment with NVS-ZP7-4 and Zn pyrithione resulted in a restoration of Cox-2 induction, and prevented induction of er stress markers and occludin. E: Increasing Zn/Pyr restored impedance loss induced by IL-1 β /TNF- α despite Zip7 inhibition with NVS-ZP7-4. The loss of impedance induced by challenge with IL-1 β /TNF- α was prevented by inclusion of NVS-ZP7-4 during the inflammatory challenge. If cytoplasmic Zn levels were restored using Zn/Pyr, impedance loss was induced by IL-1 β /TNF- α even in the presence of NVS-ZP7-4. F: The cell-permeable Zn chelator TPEN prevented overnight IL-6 induction by IL-1 β indicating intracellular Zn is essential for signaling. Cell toxicity was observed at 10 μM TPEN. G: The cell-impermeable Zn chelator DTPA had no effect on overnight IL-6 induction by IL-1 β indicating extracellular sources of Zn are not required for signaling.

We next used Zn chelators and ionophores to explore the role that altered Zn flux has in mediating the effects of Zip7 inhibition on ARPE-19 barrier function. In Zn imaging studies using the fluorescent Zn dye FluoZin3, it was found that 3–10 μM of the Zn ionophore zinc pyrithione (Zn/Pyr) caused significant Zn accumulation in the cytoplasm and ER (Fig 4A) and treatment with Zn/Pyr and NVS-ZP7-4 caused accumulation of Zn in the ER as expected (Fib. 4b). In cellular assays with Zn/Pyr, we observed full restoration of IL-6 induction by IL-1 β /TNF- α in spite of the presence of NVS-ZP7-4 (Fig 4C). Additionally, restoration of cytoplasmic Zn by Zn/Pyr prevented NVS-ZP7-4 from inhibiting Cox-2 induction and the compound was also not able to inhibit barrier dysfunction caused by IL-1 β /TNF- α (Fig 4D). Zn/Pyr also prevented the induction of occludin caused by Zip7 inhibition (S5 Fig). In cell impedance assays, inclusion of Zn/Pyr prevented the inhibition of IL-1β /TNF- α -induced barrier dysfunction by NVS-ZP7-4 (Fig 4E). The necessity of Zn for IL-1β signaling was demonstrated by the ability of the cell-permeable Zn chelator TPEN (*N*,*N*,*N′*,*N′*-tetrakis(2-pyridinylmethyl)-1,2-ethanediamine) [28] to block IL-6 induction (Fig 4F). Conversely, the cell-impermeable Zn chelator DTPA (diethylenetriaminepentaacetic acid) [28] had no effect on IL-6 induction by IL-1 β indicating that import of extracellular Zn into the cell is not required (Fig 4G). Overall, our data suggests that Zn flux from ER to cytoplasm through Zip7 is required for IL-1 β /TNF- α signaling, leading to cytokine induction and barrier dysfunction in RPE cells.

### Contribution of ER stress induced by Zip7 inhibition to maintenance of barrier function

**Fig 5:**
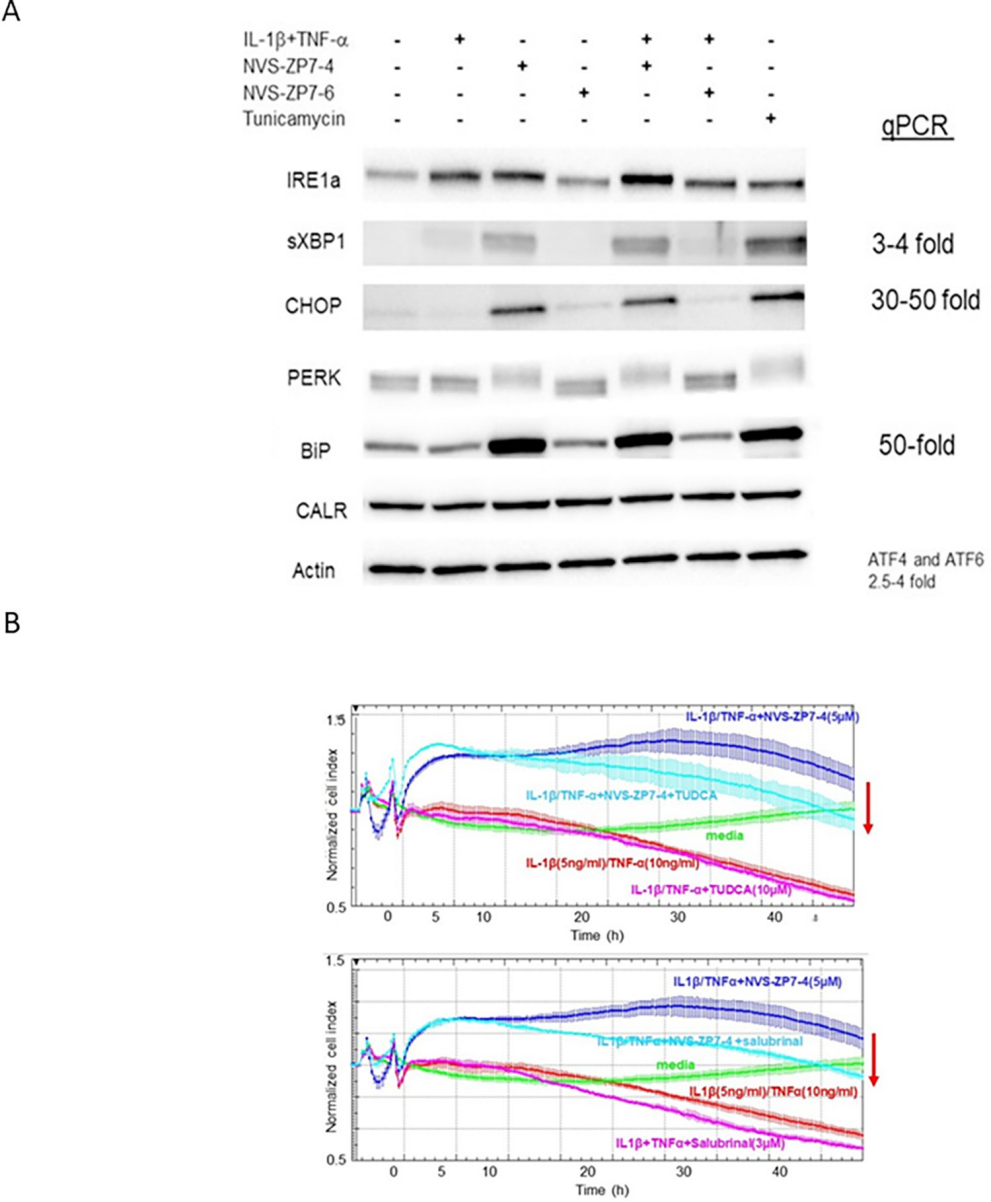
ER stress contributes to barrier protection caused by Zip7 inhibition. **A:** Cells were challenged with IL-1 β (10ng/mL)/TNF- α (20 ng/mL) in the presence or absence of NVS-ZP7-4 and NVS-ZP7-6 and tunicamycin was used as a positive control. 48h after challenge, cells were harvested for western blot analysis. NVS-ZP7-4 and tunicamycin both induced ER stress resulting in increased levels of sXBP-1, CHOP, BiP and a form of PERK with reduced gel mobility. **B:** Cells were challenged with IL-1 β (10ng/mL)/TNF- α (20 ng/mL) in the absence and presence of NVS-ZP7-4 and either 10μM TUDCA (upper) or 3μM salubrinal (lower). Zip7 inhibition with NVS-ZP7-4 prevented impedance loss induced by IL-1 β /TNF- α but inclusion of TUDCA or salubrinal with NVS-ZP7-4 during the challenge reduced impedance.

The essential role for Zn for proper signal transduction downstream of IL-1β /TNF- α suggests that Zip7 inhibition prevents barrier dysfunction through disruption of the necessary Zn flux to the cell cytoplasm. However, previous studies with Zip7 inhibitors and Zip7 knockout mice showed that prevention of Zn flux through Zip7 caused an increase in ER stress (21, 23). Consistent with this, Zip7 inhibition using NVS-ZP7-4 caused a 5-10-fold increase in levels of sXBP-1 (S6 Fig). Because Zip7 inhibition results in two outcomes: 1) reduced Zn flux into the cytoplasm and 2) increased ER stress, we wanted to try and separate the effects by inducing ER stress without altering Zn flux and seeing how an increase of ER stress alone altered the effect of IL-1 β /TNF- α in impedance assays. While challenge of cells with IL-1 β /TNF- α alone slightly increased protein levels of spliced XBP-1 (sXBP-1), treatment of cells with NVSZP7-4 alone or together with the IL-1 β /TNF- α challenge strongly increased levels of proteins associated with the ER stress response such as BiP, CHOP, sXBP-1 and a form of PERK that migrates more slowly on SDS-polyacrylamide gels which was previously shown to be associated with phosphorylation (24) whereas NVS-ZP7-6 had no effect (Fig 5A). The increase in ER stress markers caused by Zip7 inhibition were similar to those resulting from treatment with tunicamycin, a known inducer of ER stress (Fig 5A).

We then used impedance assays to test whether reduction of ER stress reduced the ability of NVS-ZP7-4 to prevent barrier dysfunction by IL-1 β /TNF- α. Treatment of cells with two compounds that decrease ER stress, the chemical chaperone tauroursodeoxycholic acid (TUDCA) [29] (Fig 5B upper) or the eIF2 α phosphatase inhibitor salubrinal [30] (Fig 5B lower), increased impedance loss by IL-1 β /TNF- α by a small amount and also partially reduced the ability of NVS-ZP7-4 to maintain barrier function in impedance assays during inflammatory challenge.

### Role of Zn flux in mediating inflammatory signaling in primary human retinal endothelial cells

**Fig 6:**
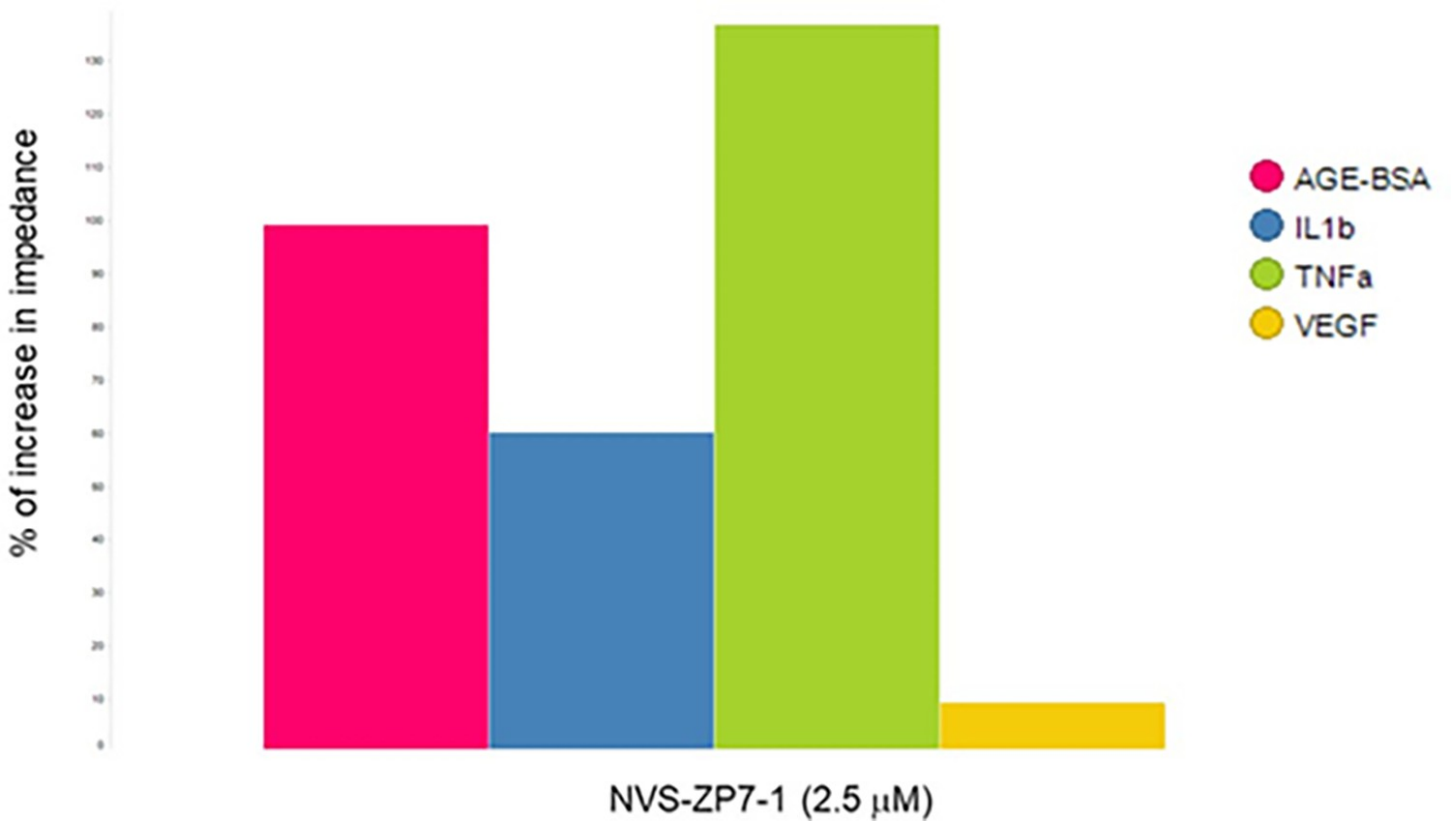
Zip7 inhibition prevents impedance loss in primary human retinal endothelial cells induced by IL-1 β, TNF- α α and glycated BSA. Impedance was measured in confluent primary human retinal endothelial cells overnight in media, media + IL-1 β (5 ng//mL), media + (TNF- α 5 ng/mL), media + AGE-BSA (250 μg/mL) and media + VEGF (50 ng/mL) in the presence and absence of NVS-ZP7-1. The % increase in impedance when compound was present versus challenge alone was calculated at 24h. Zip7 inhibition reduced impedance loss caused by IL-1 β, TNF- α and glycated BSA but not by VEGF.

In impedance assays using primary human retinal endothelial cells (HREC), NVS-ZP7-1 [23], also inhibited the effects of inflammatory signaling (IL-1β, TNF- α and glycated BSA) on barrier function of HREC (Fig 6). This data indicates that Zn requirements for inflammatory signaling pathways is not confined to RPE cells and is relevant in other cell types as well.

Overall, our results point to the key role of Zn flux from the ER to the cytoplasm for signaling downstream of IL-1β /TNF- α. Disruption of this Zn flux through Zip7 inhibition reduces the induction of inflammatory cytokines by IL-1 β /TNF- α and their ability to cause barrier disruption in RPE and HREC cells. Additionally, our data suggests that the induction of ER stress that accompanied Zip7 inhibition also exerted an anti-inflammatory effect that contributed partially to the inhibition of barrier dysfunction caused by IL-1 β /TNF- α. The ability of a Zip7 inhibitor to also block barrier dysfunction in primary retinal endothelial cells confirms the important role that Zn flux plays a fundamental role during inflammatory signaling pathways that mediate retinal barrier dysfunction. Inhibition of Zn flux may therefore be a therapeutic strategy for AMD and DME.

## Discussion

Zip7 inhibition using the small molecule NVS-ZP7-4 prevented barrier loss in TER assays using iPS-derived RPE cells challenged with TNF- α. In ARPE-19 cells, the Zip7 inhibitor NVS-ZP7-4 blocks IL-6 induction by IL-1 β and IL-1β /TNF- α as well as preventing barrier dysfunction caused by IL-1 β /TNF- α in two functional assays: impedance assays to measure conductance through a monolayer and permeability of a cell monolayer to FITC-dextran. Zip7 inhibition also prevented the reduction of protein and mRNA levels for tight junction proteins caused by IL-1 β /TNF- α.

Since NVS-ZP7-4 increases ER Zn levels and reduces cytoplasmic Zn levels as a result of blocking the flow of Zn from the ER to the cytoplasm via Zip7, the inhibitory effect on IL-1 β /TNF- α signaling strongly suggested a key role for cytoplasmic Zn in the signaling of these cytokines. This was confirmed by the inhibition of IL-6 induction by IL-1 β /TNF- α in experiments using the cell-permeable Zn chelator TPEN. The lack of effect of the cell-impermeable Zn chelator DTPA on IL-1 β /TNF- α indicates that the Zn involved in signaling is derived from intracellular sources, not extracellular. The Zn ionophore Zn/Pyr restored Zn to the cytoplasm and abrogated inhibition of IL-1 β /TNF- α by NVS-ZP7-4.

Despite the effects on inflammatory signaling by Zip7 inhibition, we were not able to measure a transient Zn spike in the cytoplasm using a Zn fluophore after cytokine stimulation. although the assay may not have been sensitive enough. There are however other links between Zip7 and inflammation. It has been reported that phosphorylation of Zip7 by casein kinase 2 (CK2) increases its activity and there is data suggesting that CK2 is activated in the TNF signaling pathway (27, 28) and during IL-1 signaling (29). It is possible therefore that active CK2 phosphorylates Zip7 and induces cytoplasmic Zn flux during IL-1 and TNF signal transduction. Other inflammatory pathways have also been shown to involve Zn flux. Zn was found necessary for proper transduction of TLR4 signaling in monocytes, during Fc receptor signaling in mast cells and downstream of IL-1 β signaling and T cell receptor activation in T cells, (14–18). Reduced phosphorylation at several nodes of the TLR4 signaling pathway suggested that Zn affected the pathway at an early step (16–18). Inhibition of a tyrosine phosphatase by Zn could potentiate tyrosine phosphorylation either by inhibiting the direct dephosphorylation of substrates or by activation of tyrosine kinases that phosphorylate substrates (30, 31). These mechanisms could alter signaling within a pathway or mediate crosstalk between pathways. For instance, inhibition of PTPRB by Zn could increase Tie2 phosphorylation and previously Tie2 phosphorylation was shown to mediate signaling that resulted in TLR4 inhibition (32). Additionally, the Tie2 pathway is of particular importance to barrier function and agonism of it has been shown to mediate barrier protection in preclinical and clinical studies (33–37). Protein tyrosine phosphorylation events have also been described during TNF- α and IL-1 signaling in other cell types, suggesting that inhibition of inhibitory tyrosine phosphatases by Zn could potentiate inflammatory pathways in RPE and retinal endothelia cells (38–41).

In addition to disrupting Zn flux in the cell, Zip7 inhibition induced ER stress. However, no evidence of ER stress was observed following incubations of cells with Zn/Pyr and NVS-ZP7-4, which suggests that ER stress occurs when cytoplasmic levels of Zn are reduced rather than when ER Zn levels are elevated. Additionally, since inhibition of Zip7 in resting cells induced markers of ER stress, this implies that a constitutive flow of Zn from the ER to the cytoplasm occurs and that blocking this flow causes ER stress. A constitutive flow of Zn from the ER through Zip7 may prevent Zn buildup in the ER or prevent depletion of cytoplasmic Zn as a result of import of cytoplasmic Zn into other cellular organelles or Zn export from the cell [31]. Since levels of Zn in the ER are significantly lower than levels observed in the cytoplasm [32], the cytoplasm seems may be more responsive to decreased Zn levels than the ER.

Since NVS-ZP7-4 blocked Zn flux from ER to cytoplasm as well as increased ER stress, both responses may contribute to the inhibition of inflammatory cytokine responses. To test this hypothesis, we used the ER stress inhibitors salubrinal and TUDCA both of which partially reduced the ability of Zip7 inhibition to block cytokine induction and barrier dysfunction caused by IL-1 β /TNF- α. This indicates that ER stress induction contributes partially to the anti-inflammatory effect of Zip7 inhibition. ER stress was previously reported to increase tight junction protein expression in RPE cells ([33] and was also found to provide short term benefits to barrier function in retinal endothelial cells [34].

Zip7 inhibition during IL-1β /TNF- α signaling can both maintains levels of mRNA encoding barrier proteins or reduce mRNA encoding cytokines or chemokines, suggesting that Zn flux affects transcriptional machinery and/or mRNA turnover. Transcriptional effects of Zn have been shown to be mediated by the Zn-dependent transcription factor MTF-1 binding metal response elements of promoters [35]. Cytoskeletal rearrangement is another mechanism by which Zn can exert effects on a signaling pathway. We observed that Zip7 inhibition reduced F-actin stress fiber formation which is consistent with studies in pulmonary endothelial cells in which a hypoxia-induced Zn flux was required for F-actin stress fiber formation [36]. Signaling downstream of IL-1, TNF- α and RAGE induce F-actin stress fibers [37–39] and stress fiber contraction compromises endothelial barrier function by disrupting cell contacts [40]. It is possible that inhibition of actin rearrangement during inflammatory signaling by reducing cytoplasmic Zn through inhibition of Zip7 is a mechanism that contributes to the broad effects of NVS-ZP7-4 on inflammatory signaling.

Our results show that reducing cytoplasmic Zn levels in ocular cells helps maintain barrier function and inhibit inflammatory signaling. In contrast, it was observed in gastrointestinal cells that cytoplasmic Zn was required to maintain barrier function and that it increased expression of claudin-3 and occludin [41]. It has also been reported to mediate inhibition of IKK leading to NfκB inhibition, findings observed both in the lung and systemically [42–44]. Therefore, the role of Zn is highly dependent on cellular context. High levels of Zn were observed in sub-RPE deposits with samples from the macula of AMD patients containing higher levels [45]. However, levels of Zn in the RPE/choroid were also found to be lower in AMD patients [46] and oral Zn supplements were found to reduce AMD progression [47–51]. It has been postulated that higher levels of Zn extracellularly and low levels intracellularly in the retina may result from RPE pathology resulting in inappropriate release of Zn to the extracellular environment [31]. Our results suggest a potential pathogenic role for Zn in the retina if levels in the cytoplasm of endothelial cells and RPE cells are dysregulated.

NVS-ZP7-4 was demonstrated in several ways to be a selective inhibitor of Zip7, It was found to bind Zip7 specifically in cellular lysates and a mutational screen identified only Zip7 mutants that reduced compound activity [23]. As a result of this specificity for Zip7, our studies indicate that Zip7 plays a key and non-redundant role in iPS-RPE and ARPE-19 cells. These studies also suggest that the er is an important source of cytoplasmic zinc for these cells that is not compensated for by Zn from other intracellular organelles nor extracellular sources. Consequently, Zn transporters involved in the import Zn to the er would also play key roles in the maintenance of Zn homeostasis in the cell.

Overall our findings suggest that Zn plays a vital role in inflammatory signaling in RPE cells and that cytoplasmic levels of Zn in these cell types is regulated by Zip7 activity. These findings suggest that modulation of cellular Zn flux can potentially be used to treat retinal disease such as AMD and DME in which inflammatory signaling contributes. The requirement of Zn for inflammatory signal transduction may be a common feature in many cell types and modulating Zn flux may be a novel approach to inhibit inflammatory responses.

## Supporting information

S1 Fig Zip7 zinc transport is involved in TNF- α signaling in RPE cells. Challenge of human iPS-derived RPE cells by TNF- α (10 ng/mL) in the presence of NVS-ZP7-4 increased transepithelial resistance relative to cells treated with DMSO alone whereas challenge with TNF- α with or without inactive NVS-ZP7-6 resulted in impedance loss.
(TIF)

S2 Fig Effect of Zip7 inhibition on mRNA levels of tight junction proteins during challenge with IL-1 β /TNF- α. qPCR analysis of several tight junction proteins were performed in ARPE-19 cells challenged with IL-1 β /TNF- α and with (NVS-ZP7-4) and without (NVS-ZP7-6) Zip7 inhibition. Zip7 inhibition during challenge resulted in an approximately 2-fold increase in CLDN-1 and ZO-1 and an approximately 4-fold increase in occludin versus mRNA levels during challenge alone.
(TIF)

S3 Fig The inhibition of Zip7 does not mediate effects on IL-1 β /TNF- α through altered Notch signaling. Confluent cells were either cultured in media or challenged with 10 ng/mL IL-1β /TNF- α in the presence or absence of the 0.04, 2.5 or 10 μM of γ-secretase inhibitor compound E (A) or dibenzazepine (DZP) (B). The impedance loss induced by IL-1β /TNF- α was not affected by treatment with DZP or **he inhibition of Zip7 does not mediate effects on IL-1** β **/TNF-** α **through altered IL-1R1 trafficking.** Confluent cells were challenged with IL-1 β in the presence or absence of NVS-ZP7-4 and the inactive analog NVS-ZP7-6. The cells were taken off the plate with EDTA and levels of IL-1R1 on unpermeabilized cells was determined by FACS analysis. No changes in cell surface levels of IL-1R1 were observed when cells were treated with IL-1 β alone or in the presence of NVS-ZP7-4 or NVS-ZP7-6.
(TIF)

S4 Fig (TIF)

S5 Fig Zn/Pyr treatment negates effect of Zip7 inhibition. Inhibition of Zip7 with NVS-ZP7-4 resulted in the induction of CHOP, occluding and xBP-1s (lane 3). These proteins were also induced by NVS-ZP7-4 in the presence of IL-1 β /TNF- α (lane 4). However, inclusion of the Zn ionophore Zn/Pyr while Zip7 was inhibited by NVS-ZP7-4 prevented upregulation of these er stress proteins.
(TIF)

S6 Fig Inhibition of Zip7 results in increased sXBP-1, a sign of ER stress. Cells were treated with NVS-ZP7-4 or NVSZP7-6 alone or with IL-1β/TNF- α At 6h, IL-1 β /TNF- α alone increased sXBP-1 approximately 2-fold, NVSZP7-4 alone increased levels of sXBP-1 approximately 5-fold and IL-1 β /TNF- α + NVS-ZP7-4 increased levels approximately 10-fold. At 30h, IL-1 β /TNF- α alone increased sXBP-1 approximately 2-fold whereas IL-1 β /TNF- α and NVS-ZP7-4 increased sXBP-1 approximately 4-fold. NVS-ZP7-6 had no effect on sXBP-1.
(TIF)

S1 Raw images Raw images for western blot analysis. Samples were prepared and western blot analysis performed as described in Methods. Pages 1–4 contain raw images for Fig 2A, pages 5–8 for Fig 3B, pages 9–17 for Fig 4D and pages 18–24 for Fig 5A.
(PDF)

S1 Data (XLSX)

S2 Data (PZFX)

S3 Data (XLSX)

S4 Data (XLSX)

S5 Data (XLSX)

S6 Data (XLSX)

S7 Data (XLSX)

S8 Data (XLSX)
