# Supplementary figures and images for: Zinc transport from the endoplasmic reticulum to the cytoplasm via Zip7 is necessary for barrier dysfunction mediated by inflammatory signaling in RPE cells
Source: PLoS One. 2022 Jul 28;17(7):e0271656. doi: 10.1371/journal.pone.0271656 (PMC9333247; doi:10.1371/journal.pone.0271656)

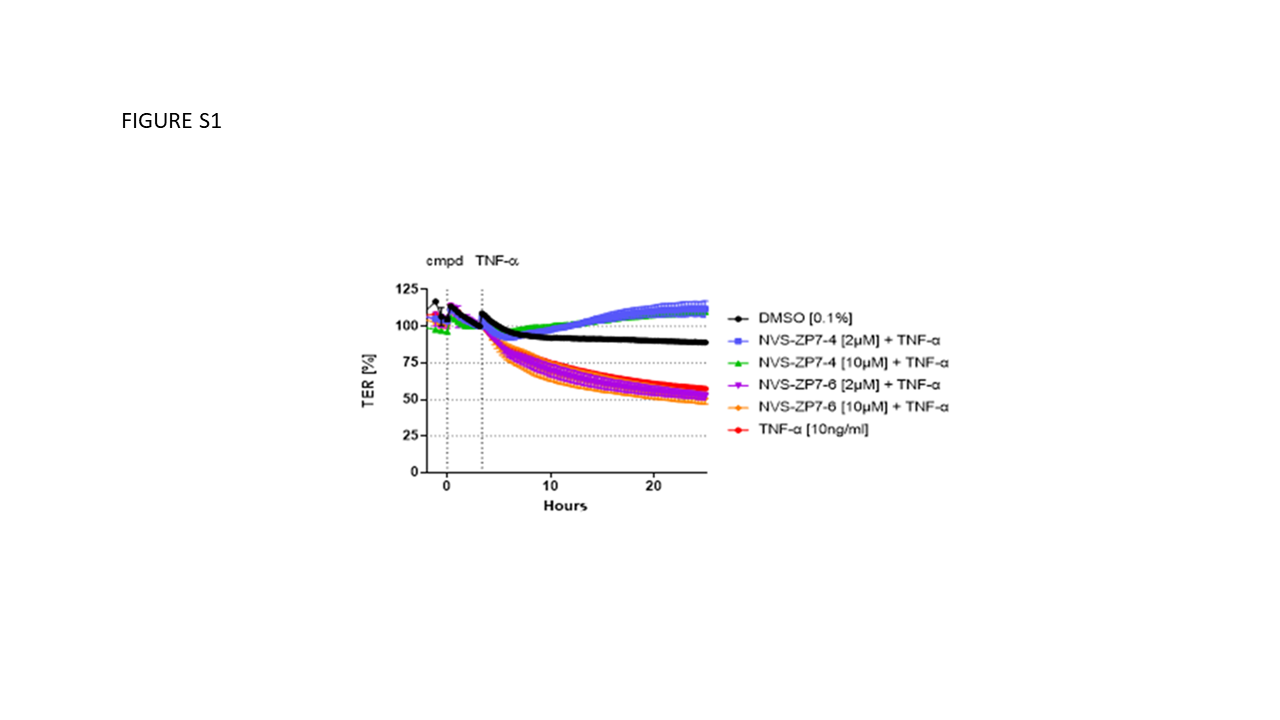

Supplement: S1 Fig — Challenge of human iPS-derived RPE cells by TNF- α (10 ng/mL) in the presence of NVS-ZP7-4 increased transepithelial resistance relative to cells treated with DMSO alone whereas challenge with TNF- α with or without inactive NVS-ZP7-6 resulted in impedance loss. (TIF) [file pone.0271656.s001.tif]

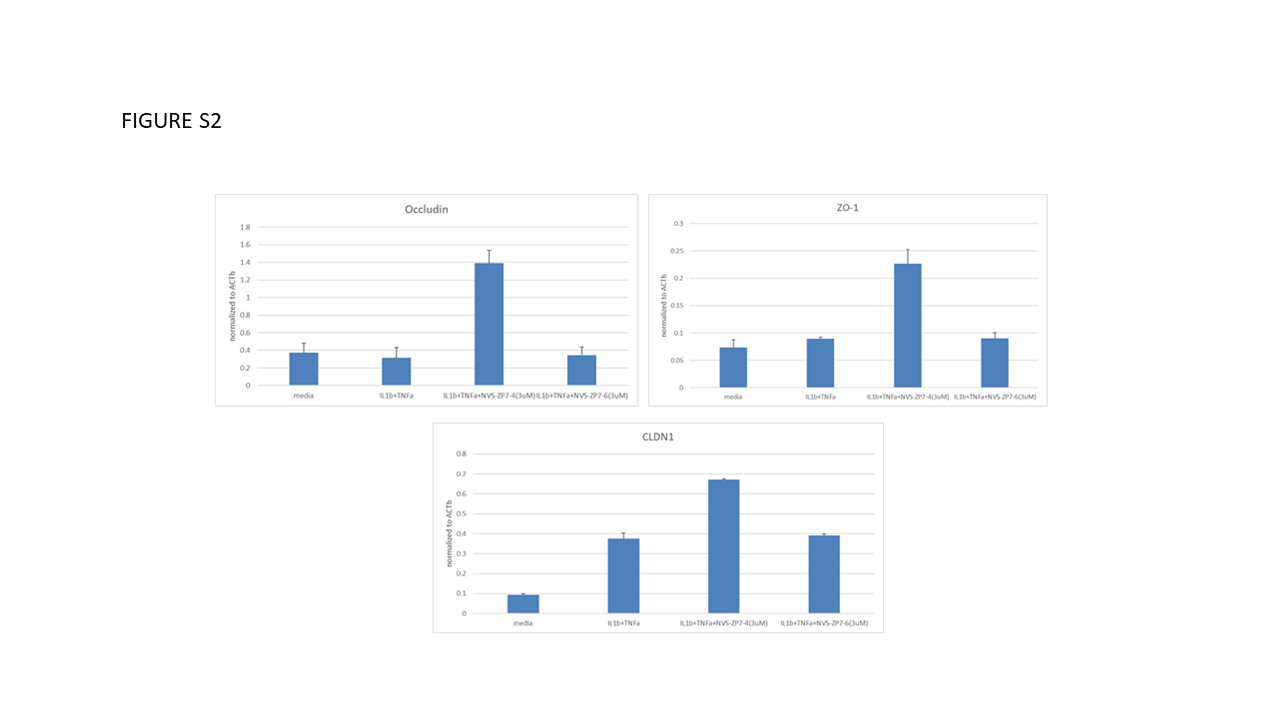

Supplement: S2 Fig — Zip7 inhibition during challenge resulted in an approximately 2-fold increase in CLDN-1 and ZO-1 and an approximately 4-fold increase in occludin versus mRNA levels during challenge alone. (TIF) [file pone.0271656.s002.tif]

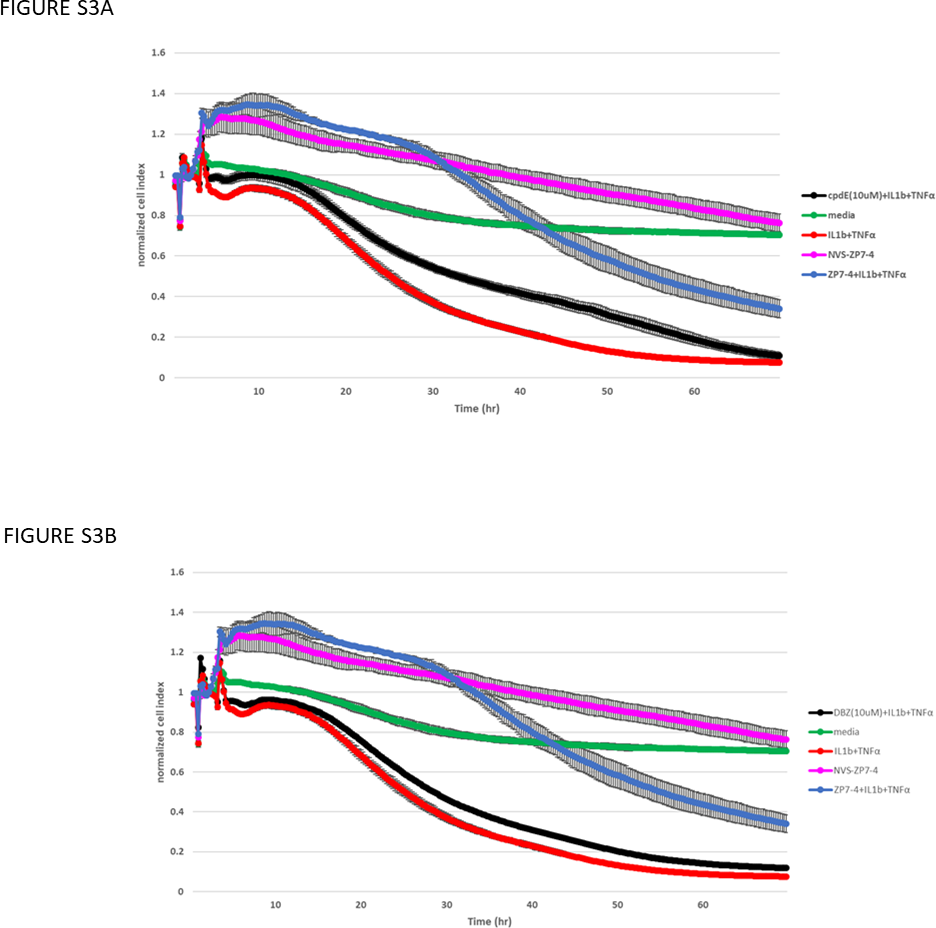

Supplement: S3 Fig — Confluent cells were either cultured in media or challenged with 10 ng/mL IL-1β /TNF- α in the presence or absence of the 0.04, 2.5 or 10 μM of γ-secretase inhibitor compound E (A) or dibenzazepine (DZP) (B). The impedance loss induced by IL-1β /TNF- α was not affected by treatment with DZP or he inhibition of Zip7 does not mediate effects on IL-1 β /TNF- α through altered IL-1R1 trafficking. Confluent cells were challenged with IL-1 β in the presence or absence of NVS-ZP7-4 and the inactive analog NVS-ZP7-6. The cells were taken off the plate with EDTA and levels of IL-1R1 on unpermeabilized cells was determined by FACS analysis. No changes in cell surface levels of IL-1R1 were observed when cells were treated with IL-1 β alone or in the presence of NVS-ZP7-4 or NVS-ZP7-6. (TIF) [file pone.0271656.s003.tif]

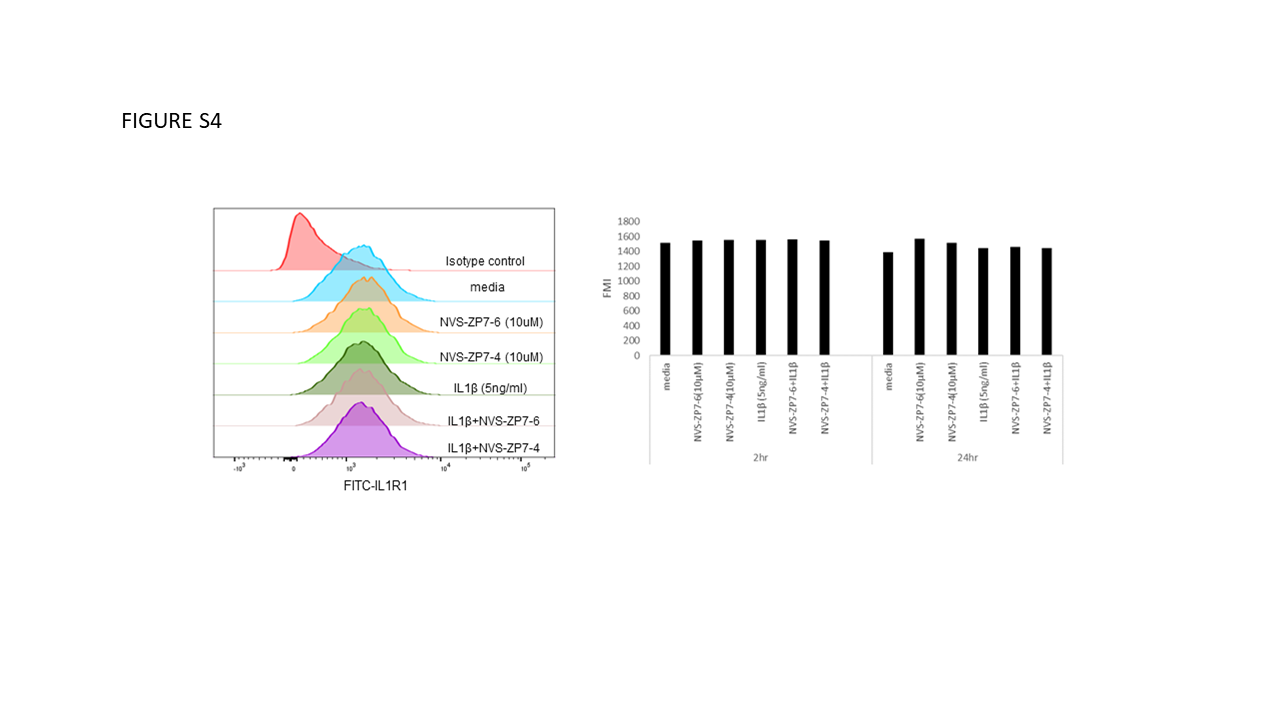

Supplement: S4 Fig — (TIF) [file pone.0271656.s004.tif]

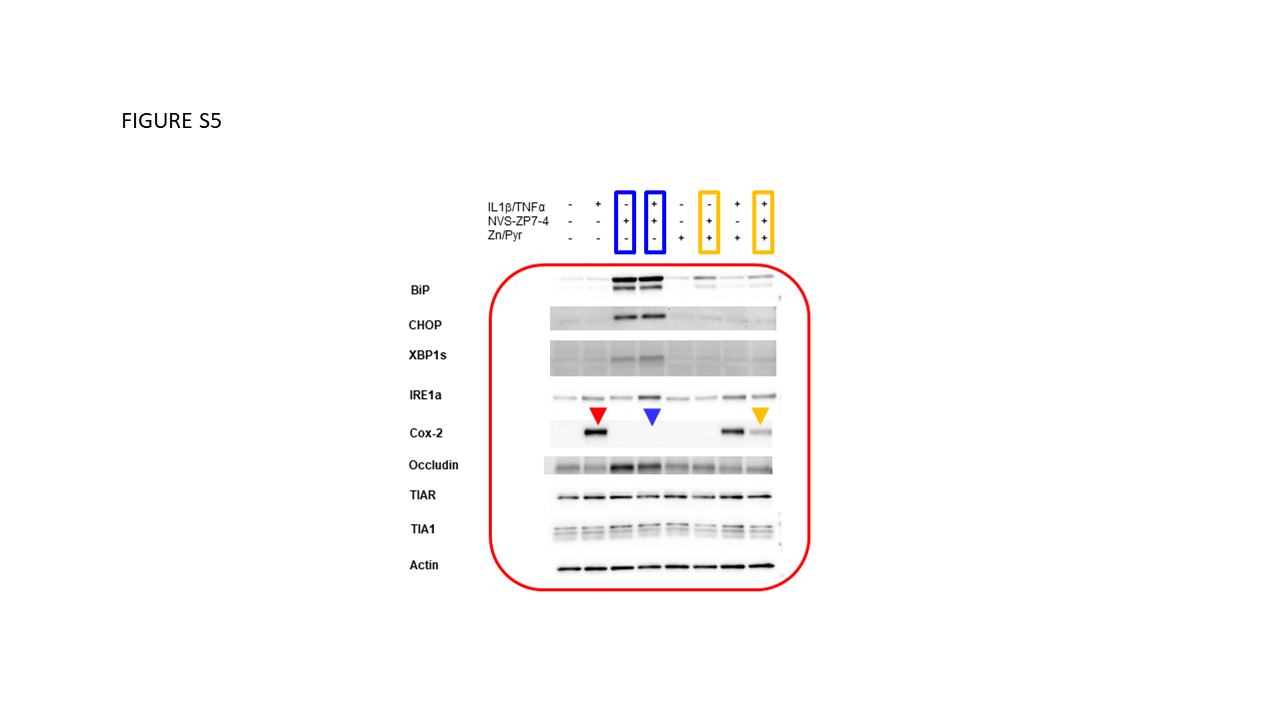

Supplement: S5 Fig — Inhibition of Zip7 with NVS-ZP7-4 resulted in the induction of CHOP, occluding and xBP-1s (lane 3). These proteins were also induced by NVS-ZP7-4 in the presence of IL-1 β /TNF- α (lane 4). However, inclusion of the Zn ionophore Zn/Pyr while Zip7 was inhibited by NVS-ZP7-4 prevented upregulation of these er stress proteins. (TIF) [file pone.0271656.s005.tif]

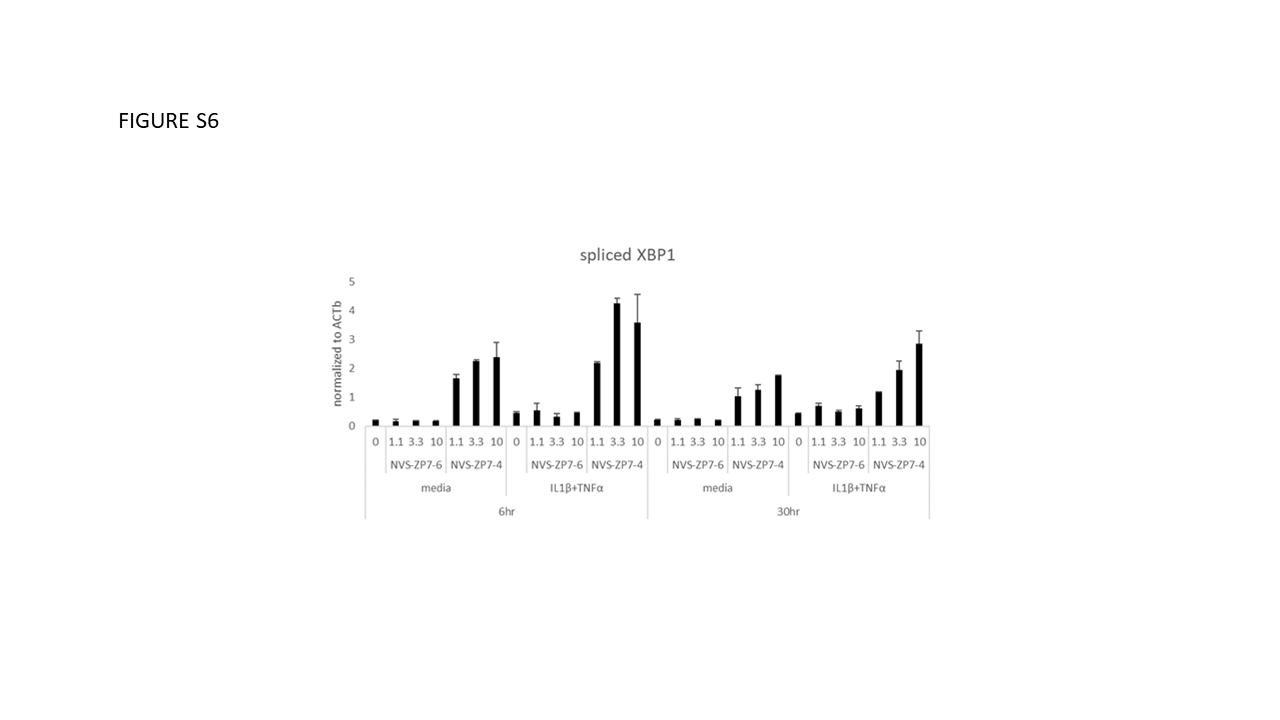

Supplement: S6 Fig — Cells were treated with NVS-ZP7-4 or NVSZP7-6 alone or with IL-1β/TNF- α At 6h, IL-1 β /TNF- α alone increased sXBP-1 approximately 2-fold, NVSZP7-4 alone increased levels of sXBP-1 approximately 5-fold and IL-1 β /TNF- α + NVS-ZP7-4 increased levels approximately 10-fold. At 30h, IL-1 β /TNF- α alone increased sXBP-1 approximately 2-fold whereas IL-1 β /TNF- α and NVS-ZP7-4 increased sXBP-1 approximately 4-fold. NVS-ZP7-6 had no effect on sXBP-1. (TIF) [file pone.0271656.s006.tif]
