# Supplementary material for: Zinc transport from the endoplasmic reticulum to the cytoplasm via Zip7 is necessary for barrier dysfunction mediated by inflammatory signaling in RPE cells
Source: PLoS One. 2022 Jul 28;17(7):e0271656. doi: 10.1371/journal.pone.0271656 (PMC9333247; doi:10.1371/journal.pone.0271656)
Supplement: S1 Raw images — Samples were prepared and western blot analysis performed as described in Methods. Pages 1–4 contain raw images for Fig 2A, pages 5–8 for Fig 3B, pages 9–17 for Fig 4D and pages 18–24 for Fig 5A. (PDF) [file pone.0271656.s007.pdf]

Fig2A

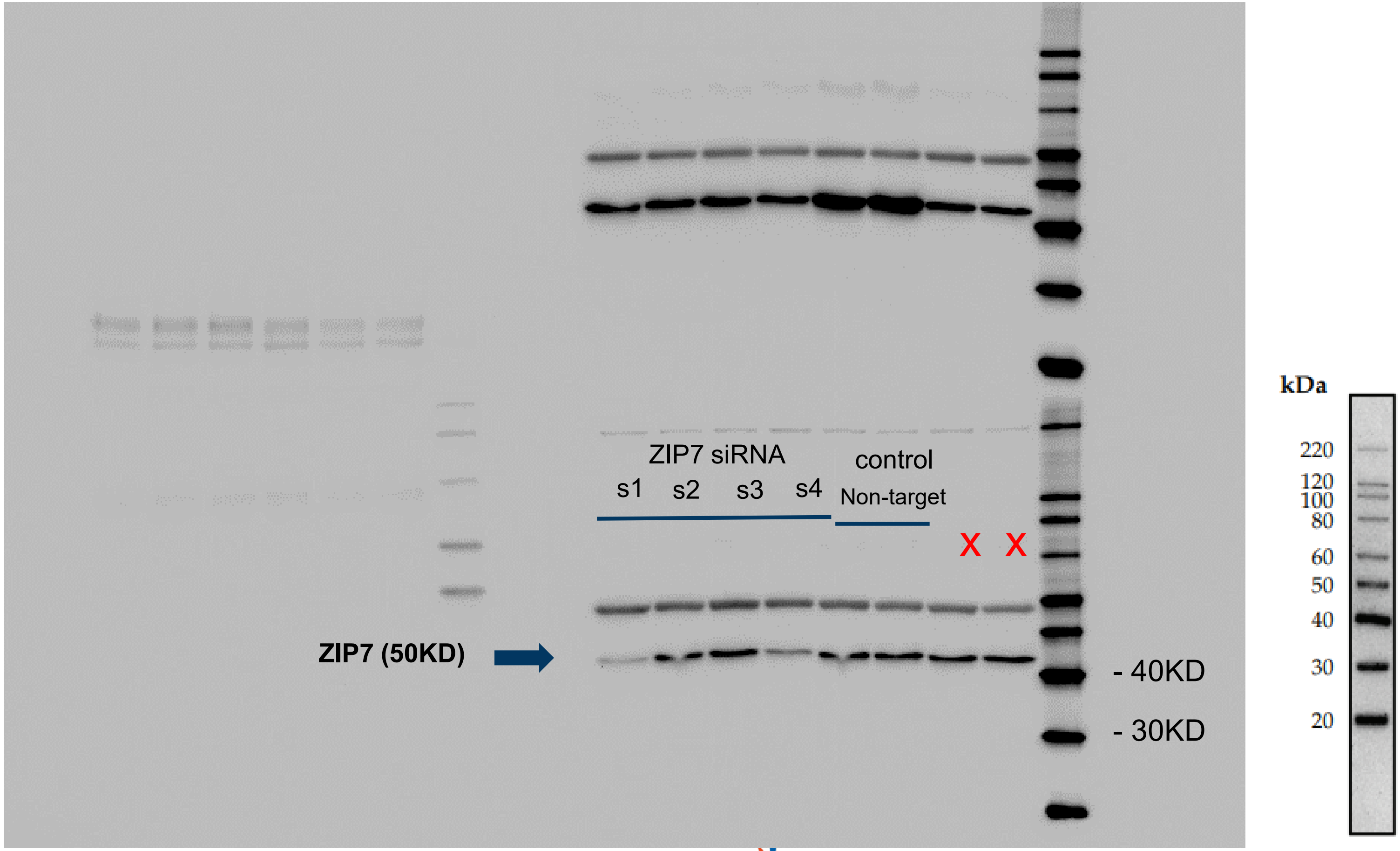

NOVARTIS | Reimagining Medicine

Fig2A

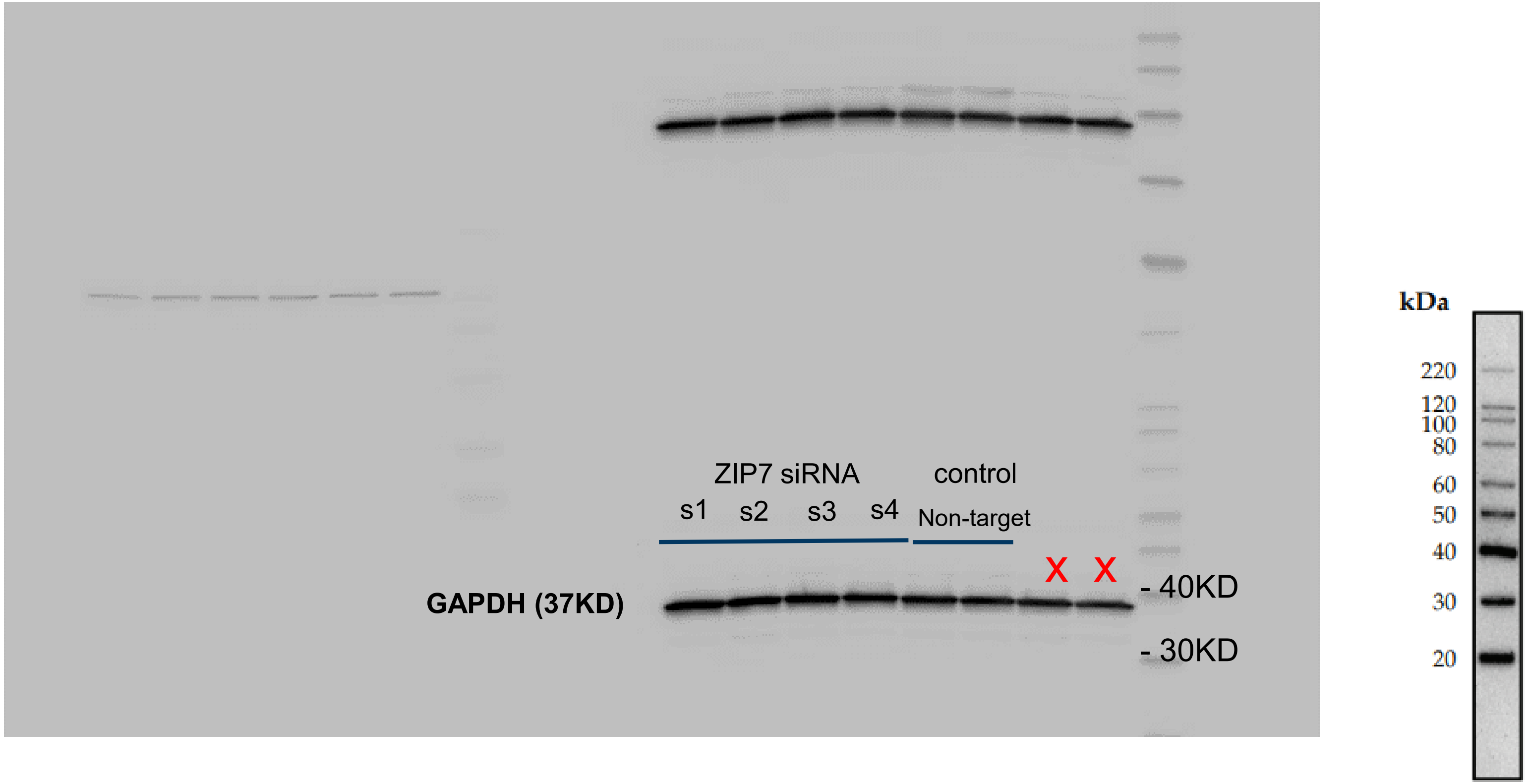

NOVARTIS | Reimagining Medicine

Fig2A

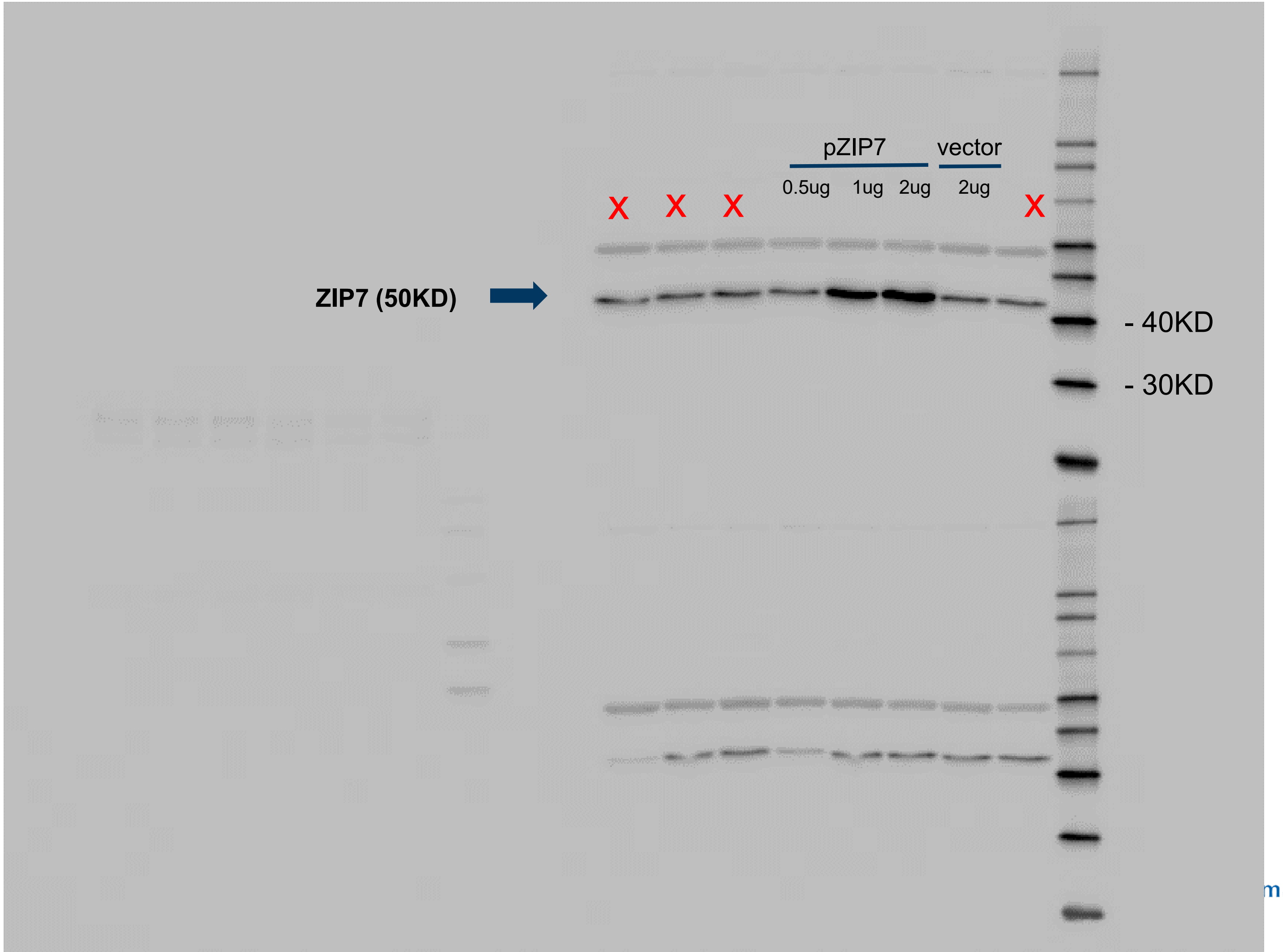

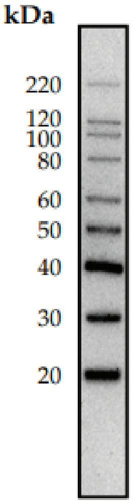

magining Medicine

Fig2A

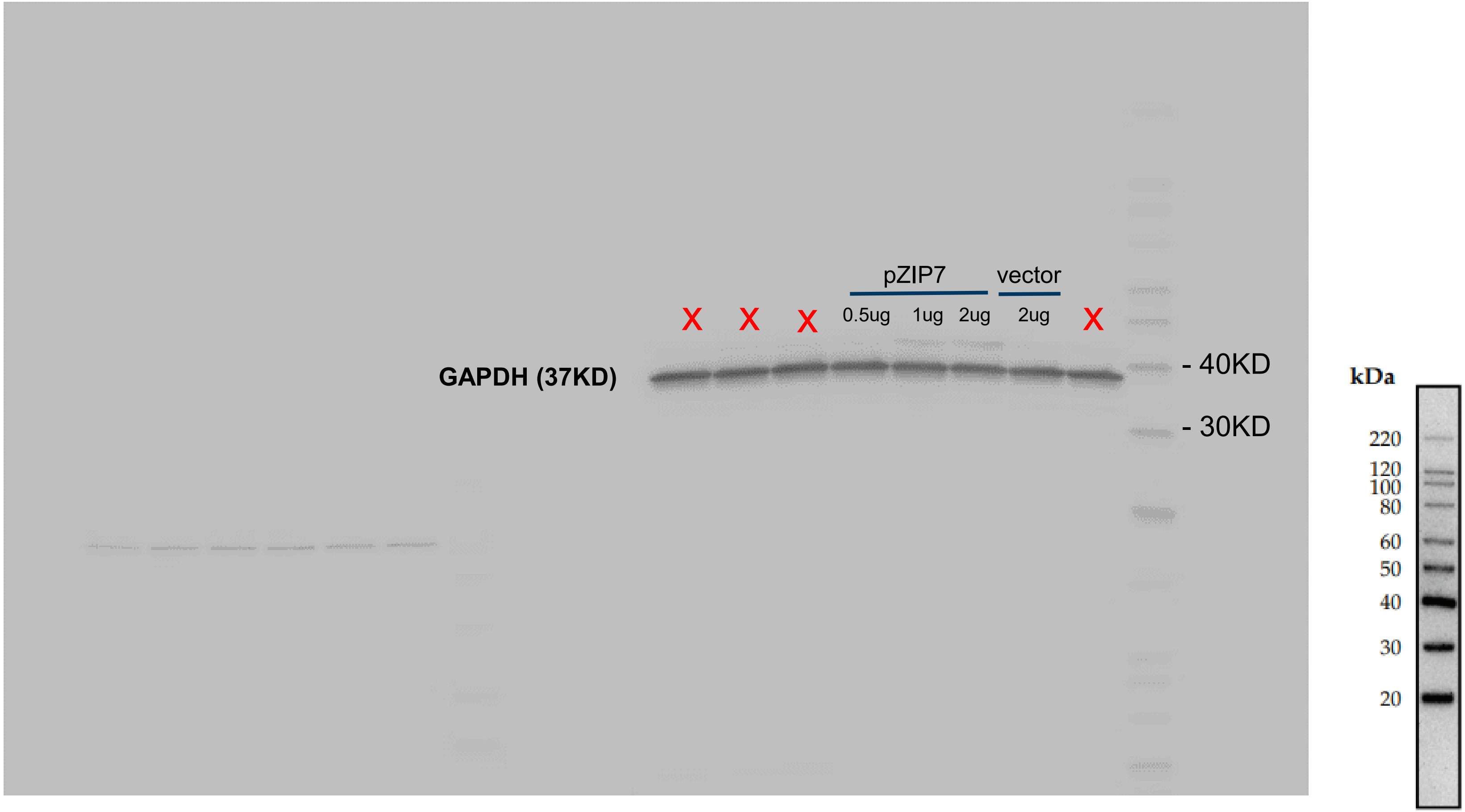

NOVARTIS | Reimagining Medicine

Fig 3B

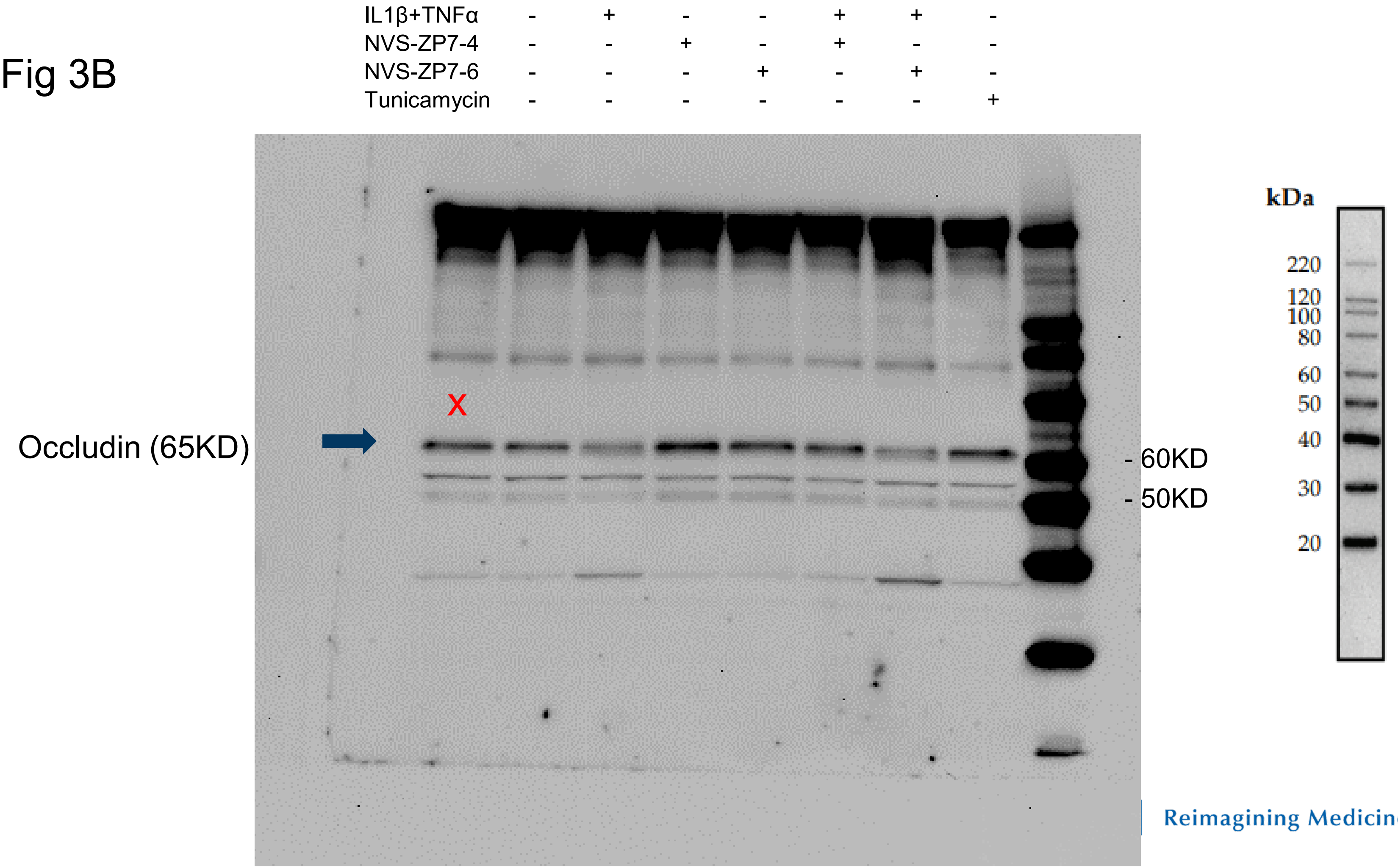

IL1β+TNFα - + - - + + -
NVS-ZP7-4 - - + - + - -
NVS-ZP7-6 - - - + - + -
Tunicamycin - - - - - - +
X
Occludin (65KD)
- 60KD
- 50KD
kDa
220
120
100
80
60
50
40
30
20

Fig 3B

IL1β+TNFα	-	+	-	-	+	+	-
NVS-ZP7-4	-	-	+	-	+	-	-
NVS-ZP7-6	-	-	-	+	-	+	-
Tunicamycin	-	-	-	-	-	-	+

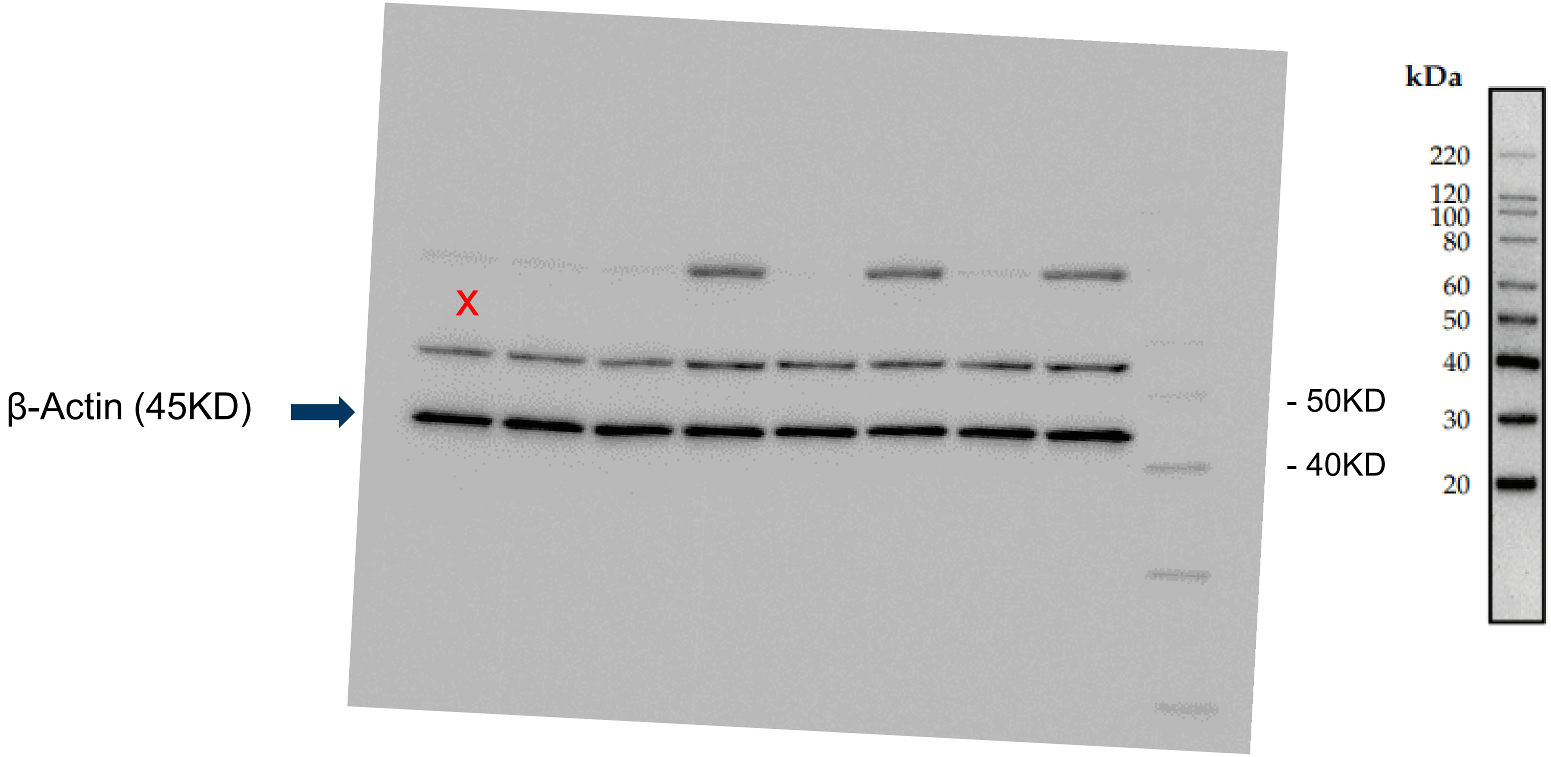

Fig 3B

IL1β+TNFα	-	+	-	-	+	+	-
NVS-ZP7-4	-	-	+	-	+	-	-
NVS-ZP7-6	-	-	-	+	-	+	-
Tunicamycin	-	-	-	-	-	-	+

Cox2 (74KD)

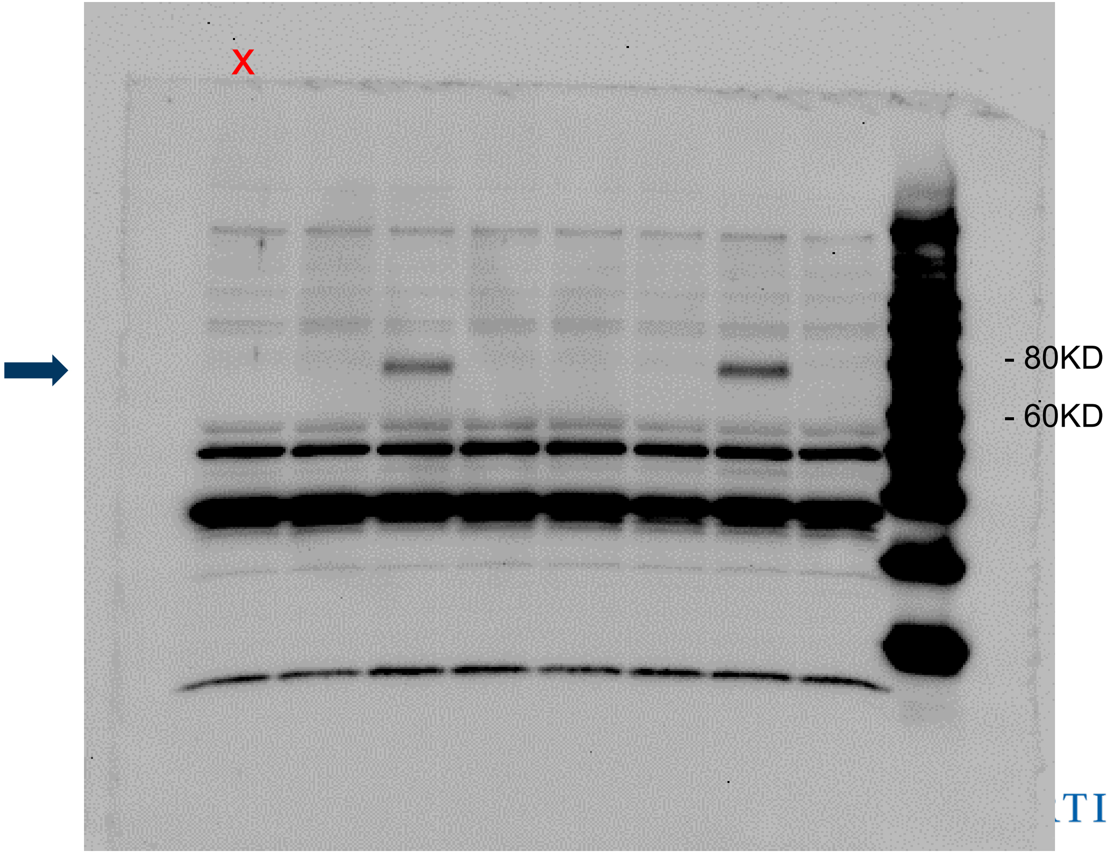

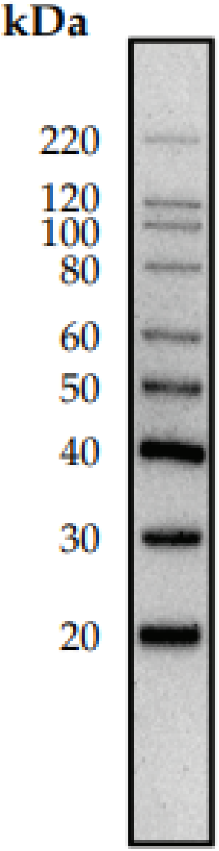

Fig 3B

IL1β+TNFα	-	+	-	-	+	+	-
NVS-ZP7-4	-	-	+	-	+	-	-
NVS-ZP7-6	-	-	-	+	-	+	-
Tunicamycin	-	-	-	-	-	-	+

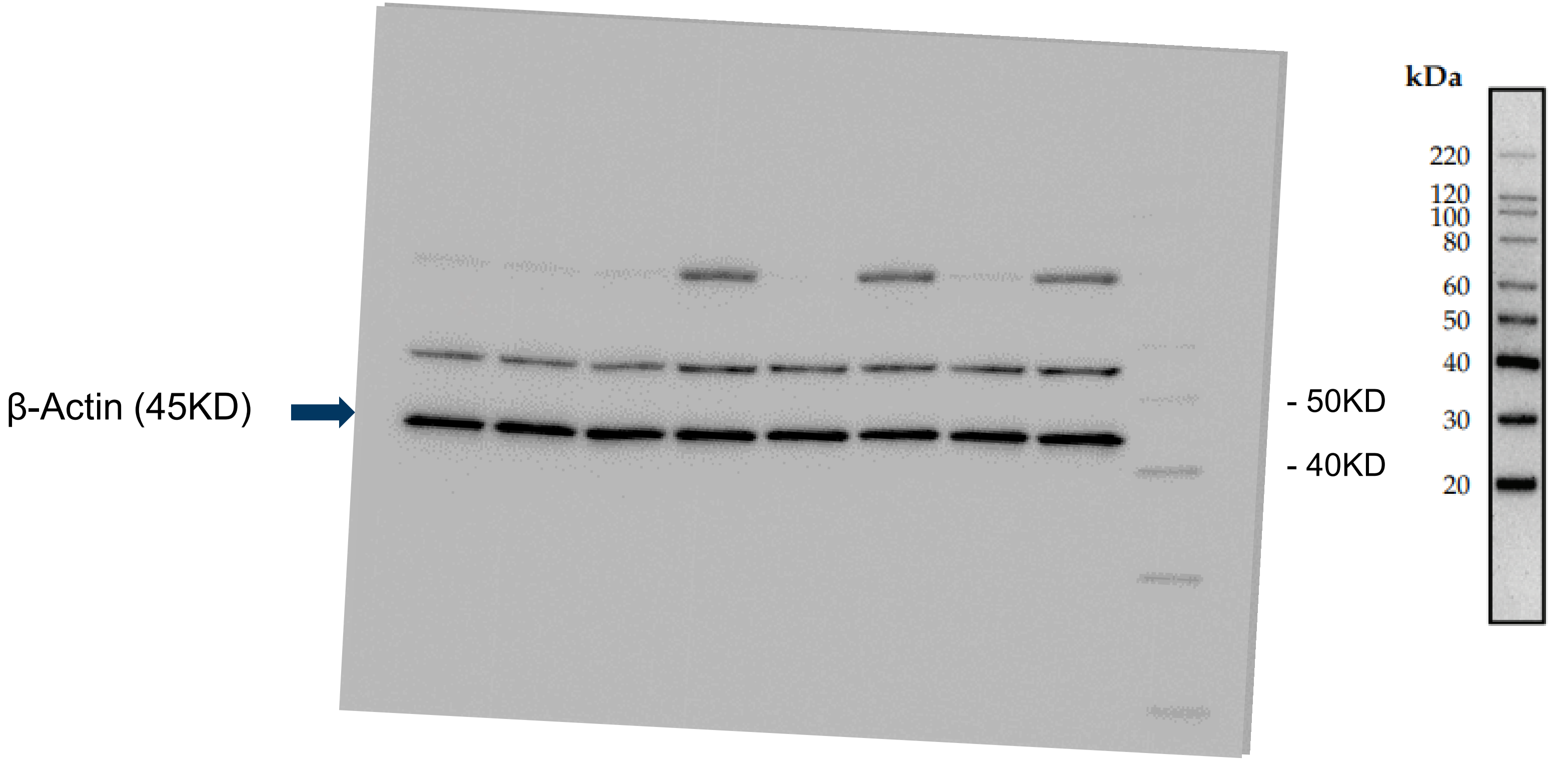

NOVARTIS | Reimagining Medicine

Fig 4D

IL1β/TNFα	-	+	-	+	-	-	+	+
NVS-ZP7-4	-	-	+	+	-	+	-	+
Zn/Pyr	-	-	-	-	+	+	+	+

BiP (78KD)

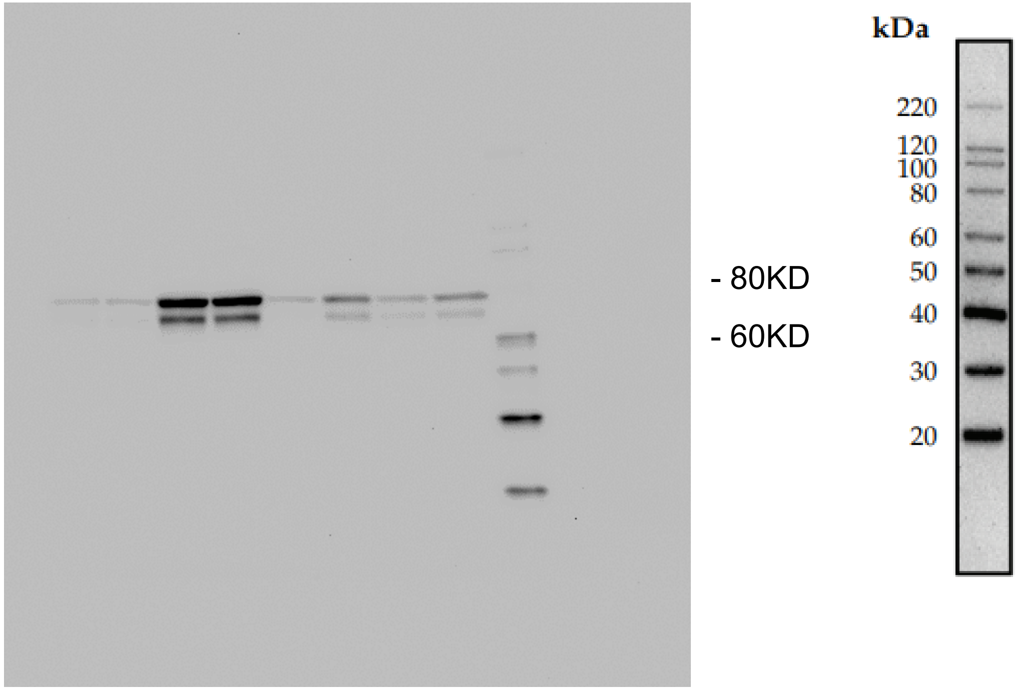

NOVARTIS | Reimagining Medicine

Fig 4D

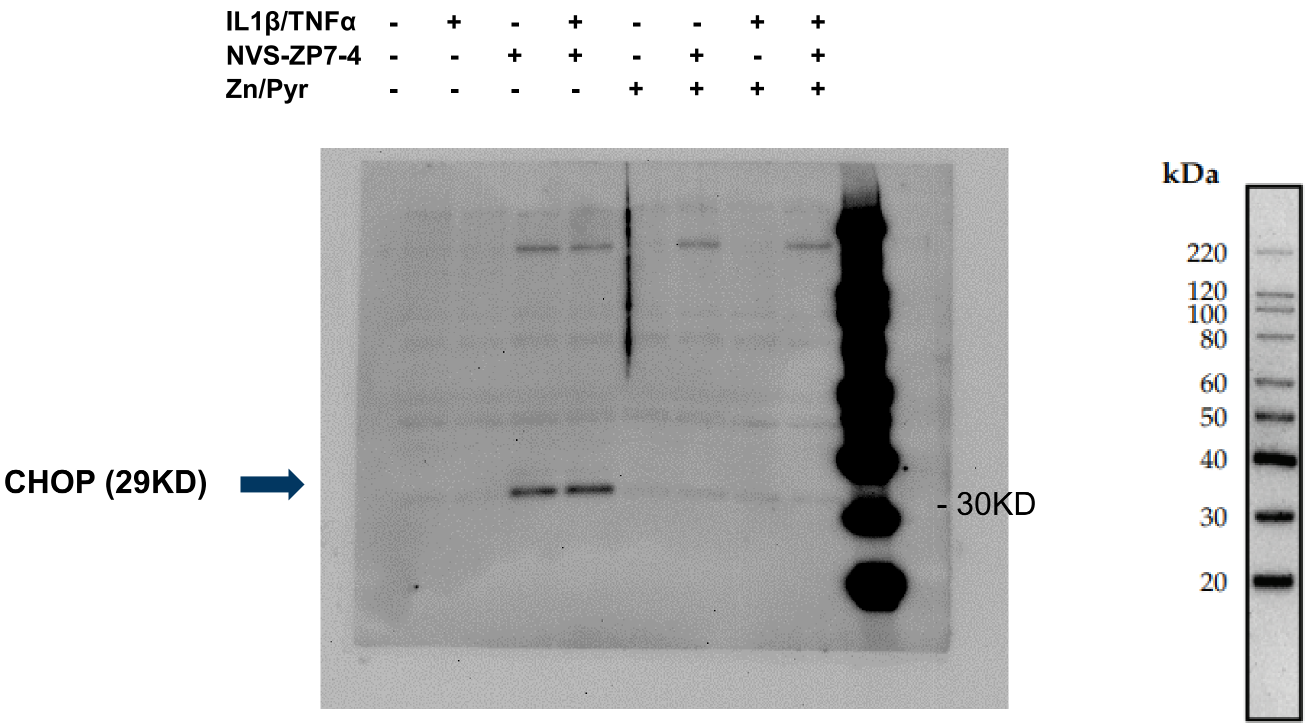
IL1β/TNFα - + - + - - + +
NVS-ZP7-4 - - + + - + - +
Zn/Pyr - - - - + + + +
CHOP (29KD)
- 30KD
kDa
220
120
100
80
60
50
40
30
20

Fig 4D

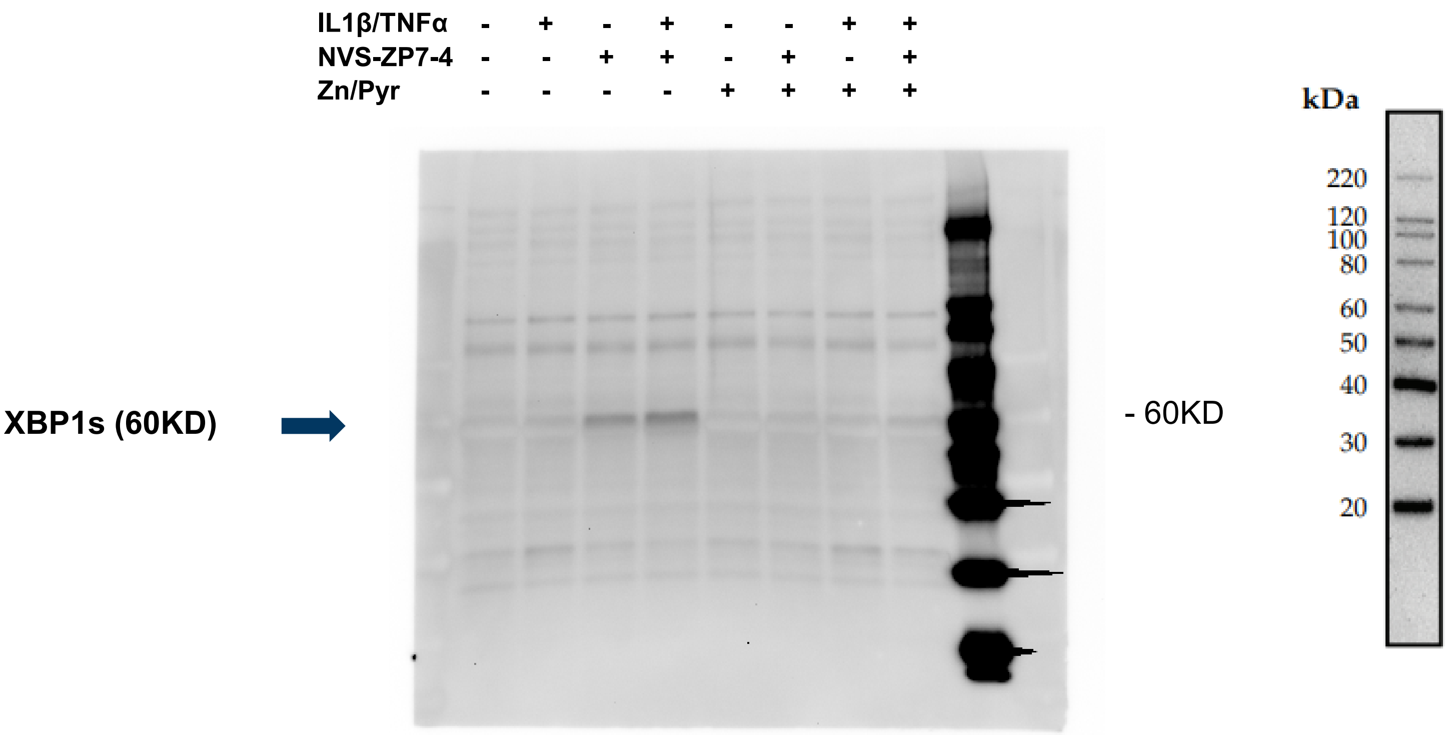

NOVARTIS | Reimagining Medicine

Fig 4D

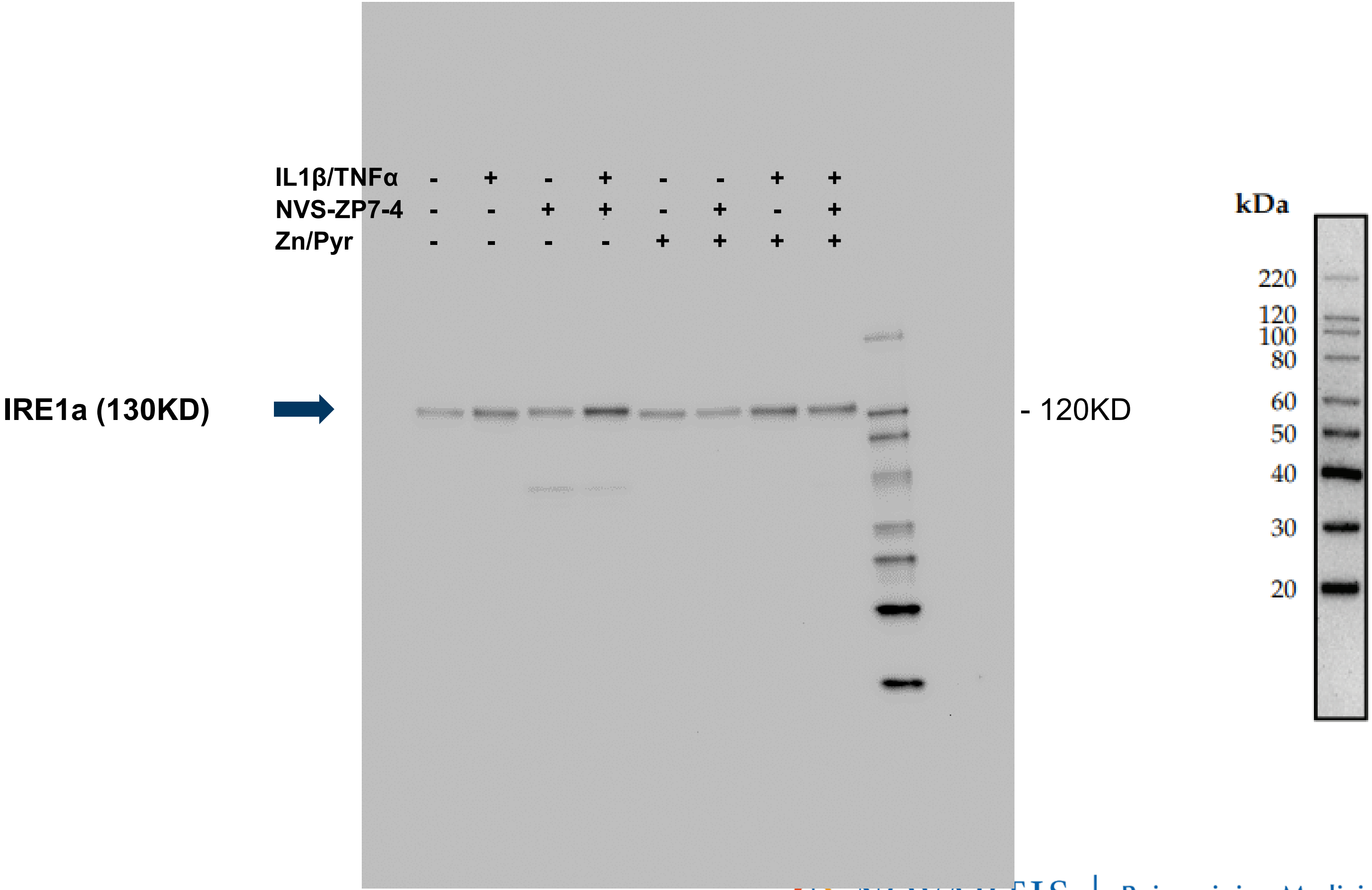

NOVARTIS | Reimagining Medicine

Fig 4D

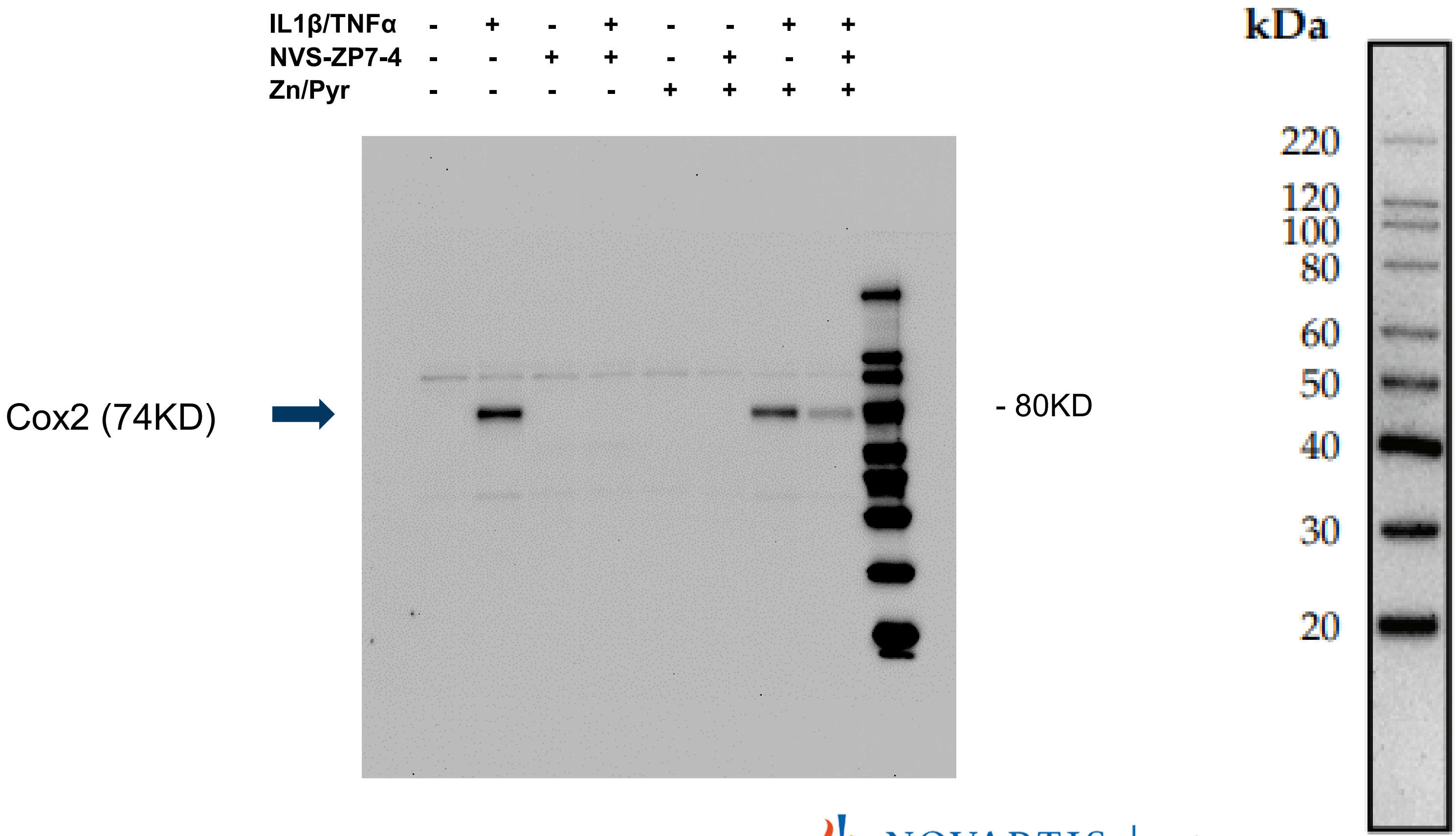
IL1β/TNFα - + - + - - + +
NVS-ZP7-4 - - + + - + - +
Zn/Pyr - - - - + + + +
Cox2 (74KD)
- 80KD
kDa
220
120
100
80
60
50
40
30
20

Fig 4D

IL1β/TNFα	-	+	-	+	-	-	+	+
NVS-ZP7-4	-	-	+	+	-	+	-	+
Zn/Pyr	-	-	-	-	+	+	+	+

Occludin (65KD)

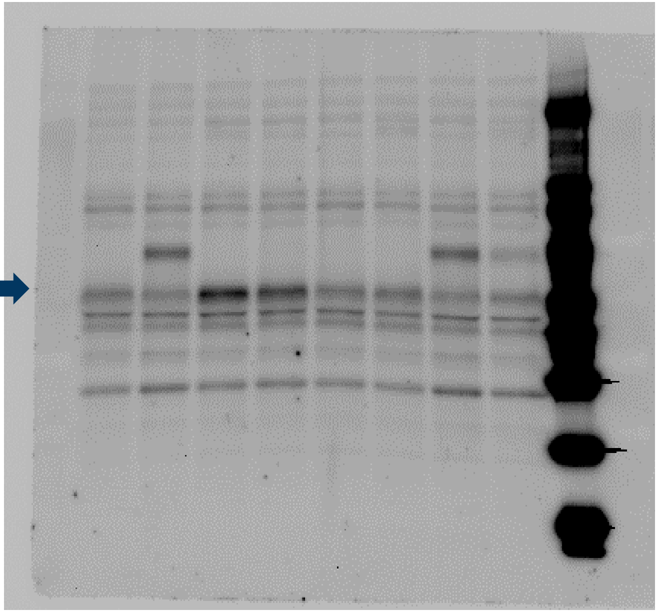

- 60KD

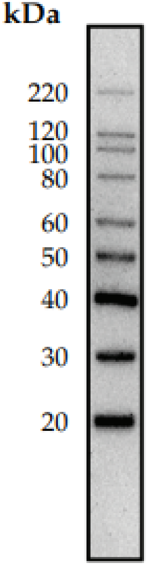

NOVARTIS | Reimagining Medicine

Fig 4D

IL1β/TNFα	-	+	-	+	-	-	+	+
NVS-ZP7-4	-	-	+	+	-	+	-	+
Zn/Pyr	-	-	-	-	+	+	+	+

TIAR (42KD)

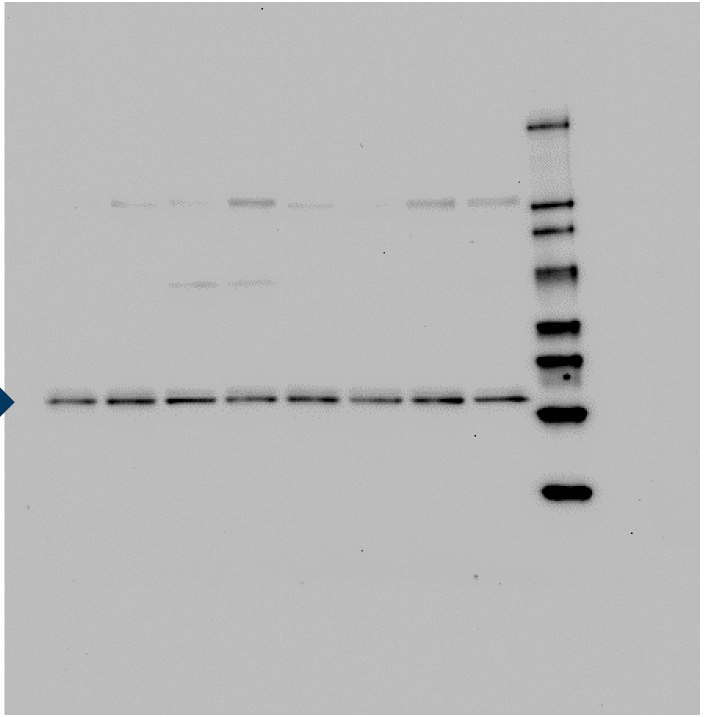

- 40KD

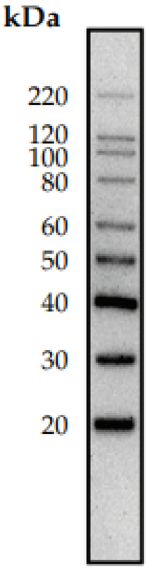

NOVARTIS | Reimagining Medicine

Fig 4D

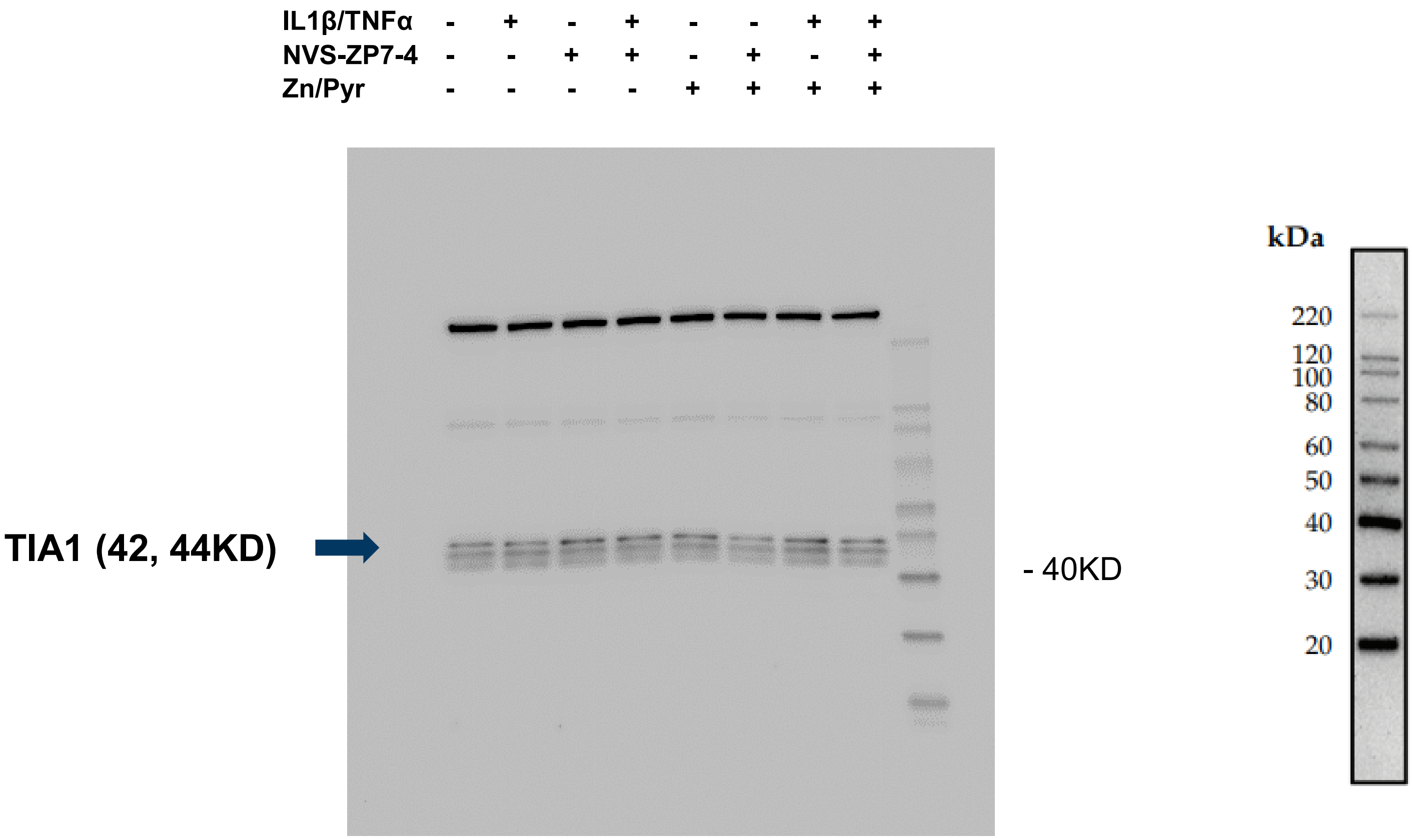

NOVARTIS | Reimagining Medicine

Fig 4D

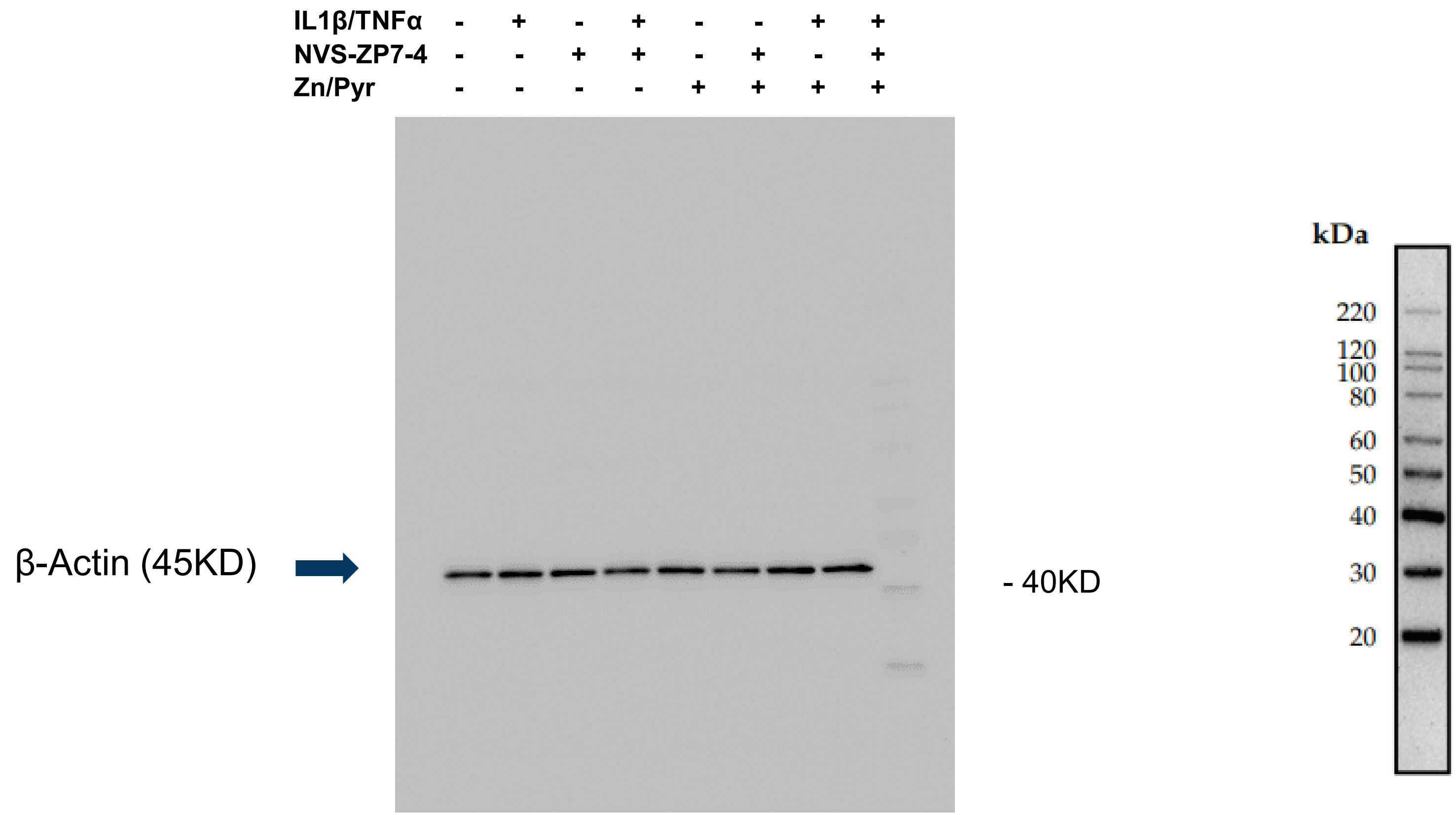

NOVARTIS | Reimagining Medicine

Fig 5A

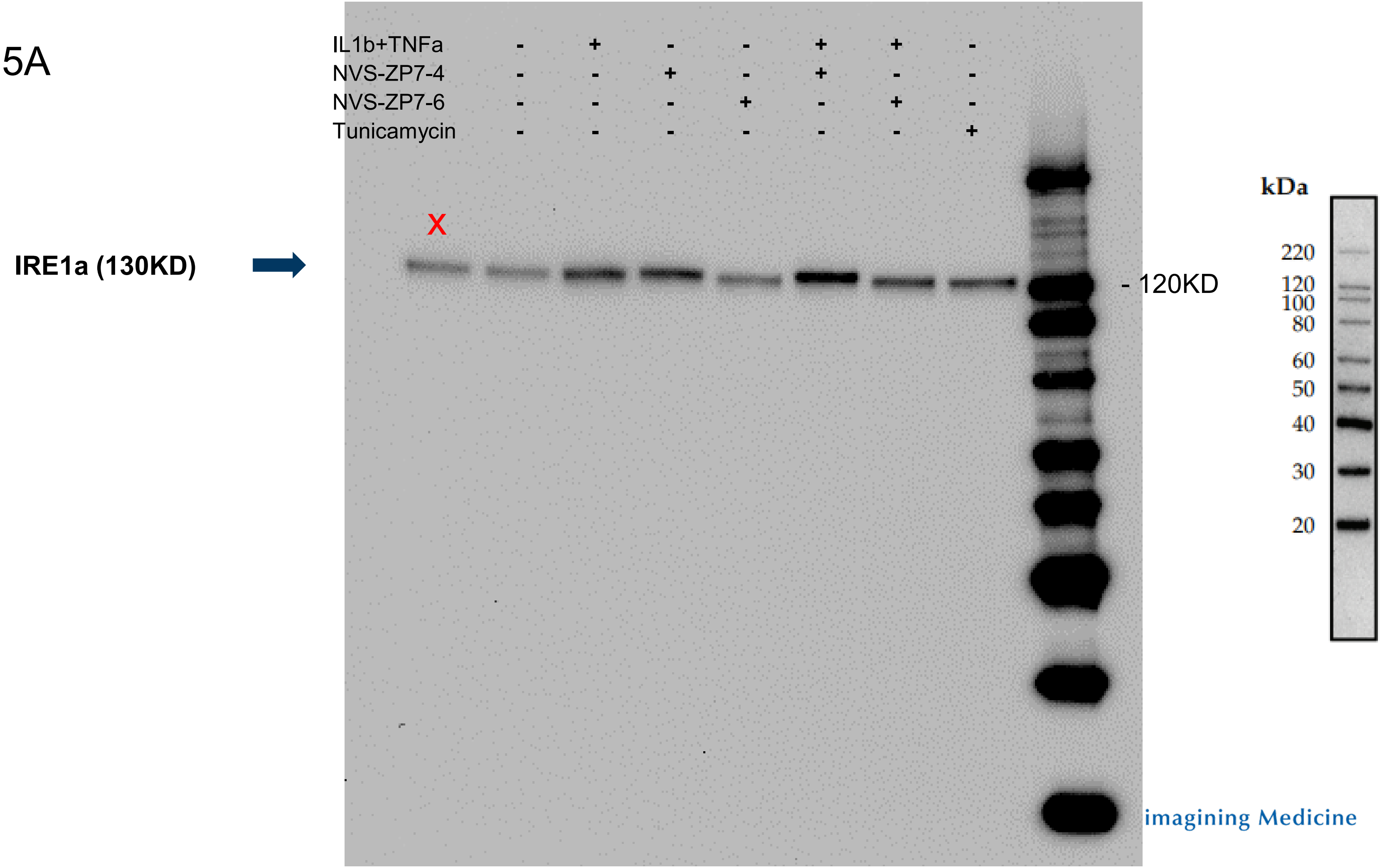

imagining Medicine

Fig 5A

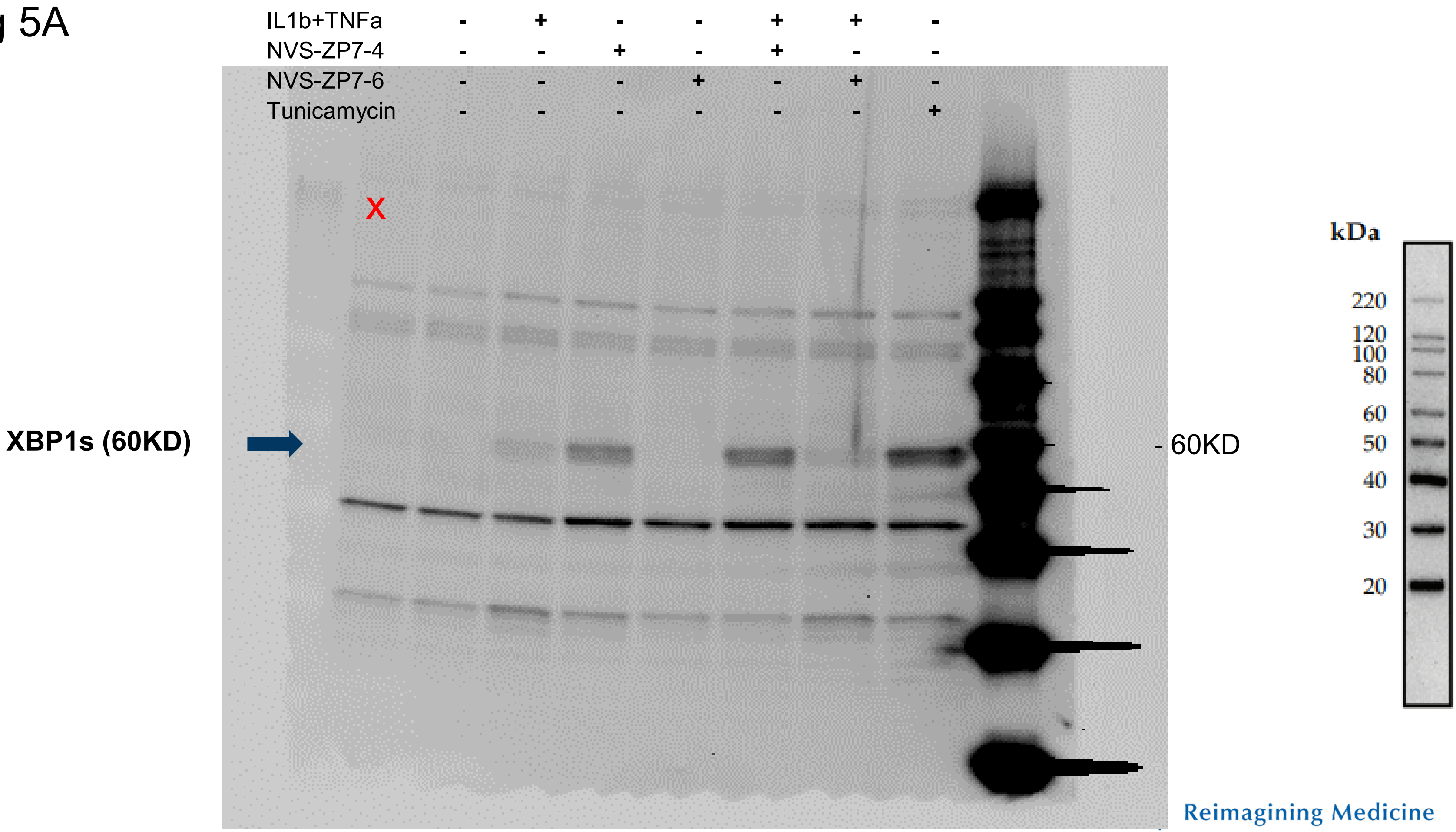

IL1b+TNFa - + - - + + -
NVS-ZP7-4 - - + - + - -
NVS-ZP7-6 - - - + - + -
Tunicamycin - - - - - - +
X
XBP1s (60KD)
- 60KD
kDa
220
120
100
80
60
50
40
30
20

Fig 5A

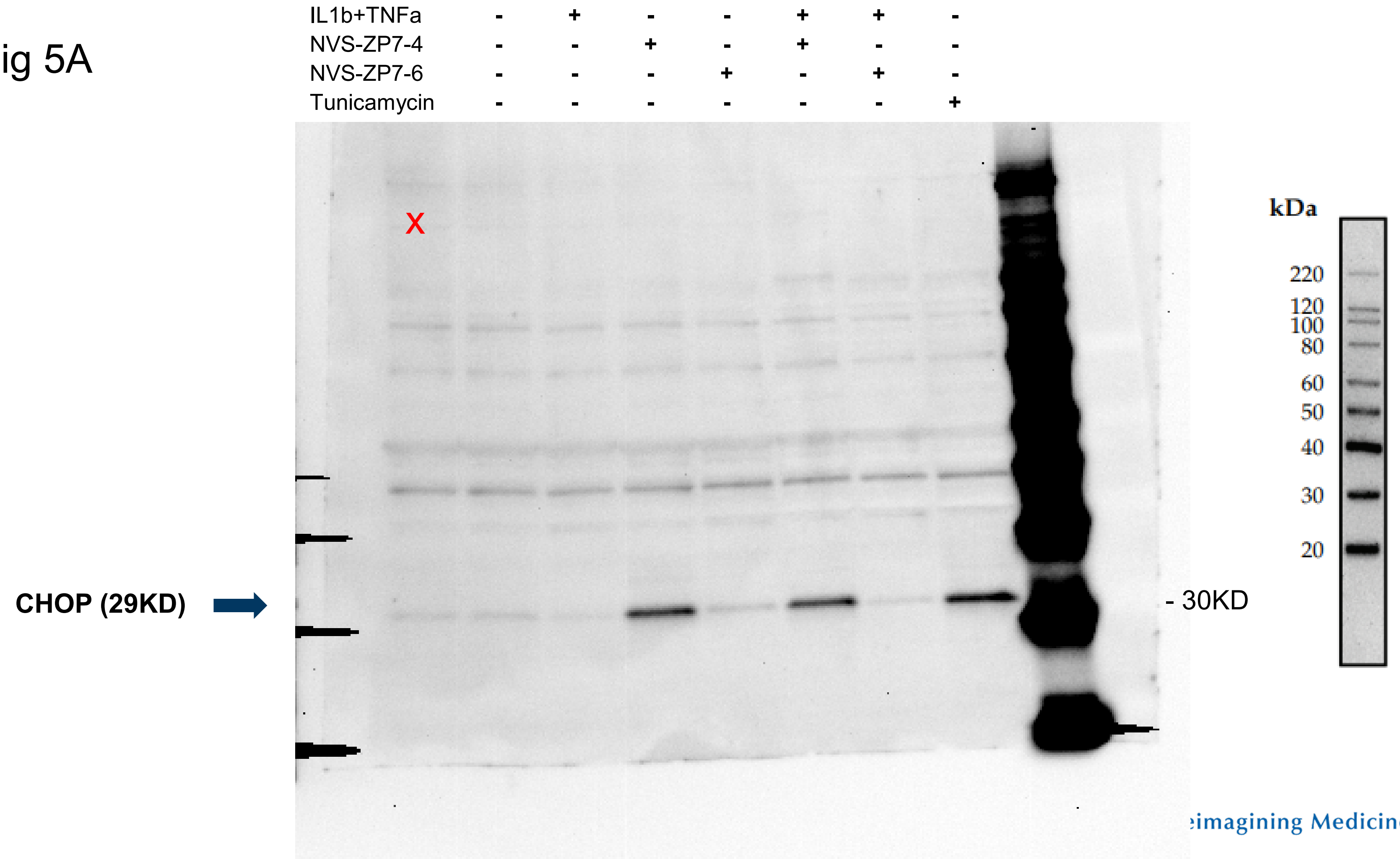

ːimagining Medicine

Fig 5A

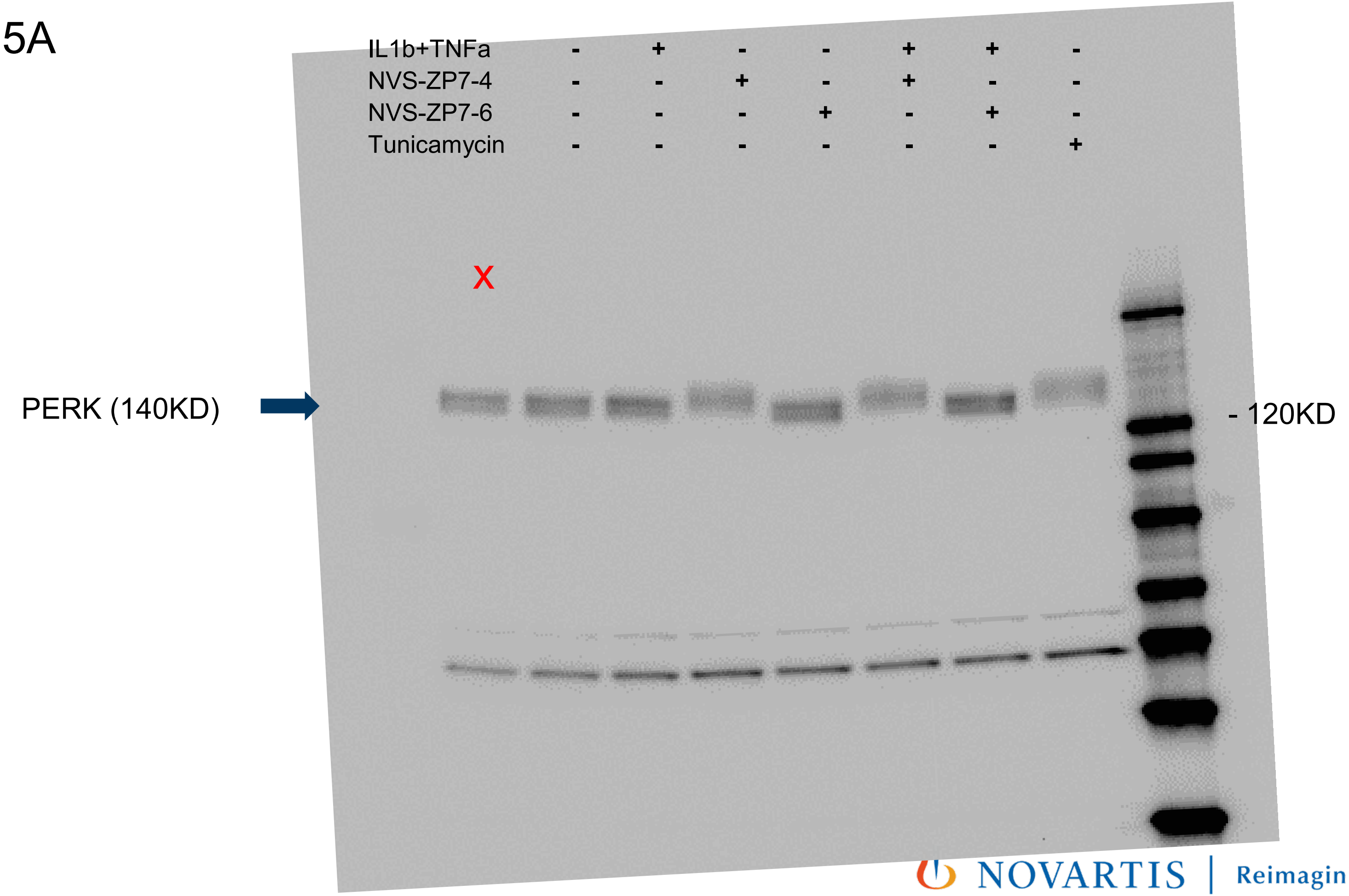
IL1b+TNFa - + - - + + -
NVS-ZP7-4 - - + - + - -
NVS-ZP7-6 - - - + - + -
Tunicamycin - - - - - - +
X
PERK (140KD)
- 120KD

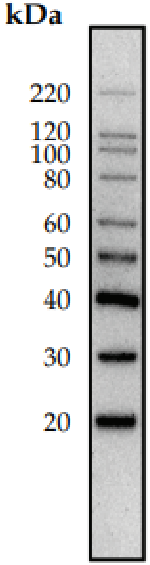
kDa
220
120
100
80
60
50
40
30
20

Fig 5A

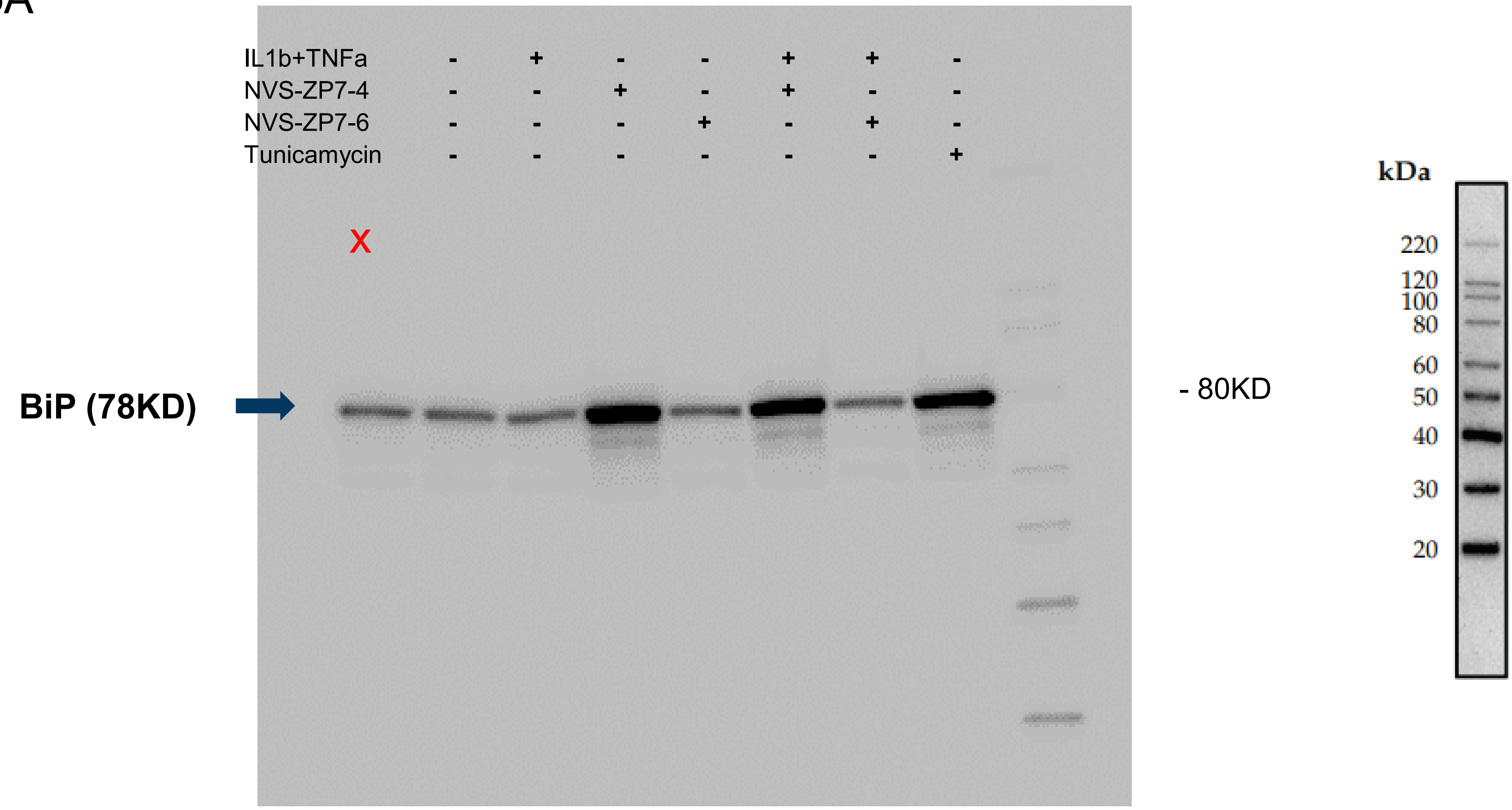
IL1b+TNFa - + - - + + -
NVS-ZP7-4 - - + - + - -
NVS-ZP7-6 - - - + - + -
Tunicamycin - - - - - - +
X
BiP (78KD)
- 80KD
kDa
220
120
100
80
60
50
40
30
20

Fig 5A

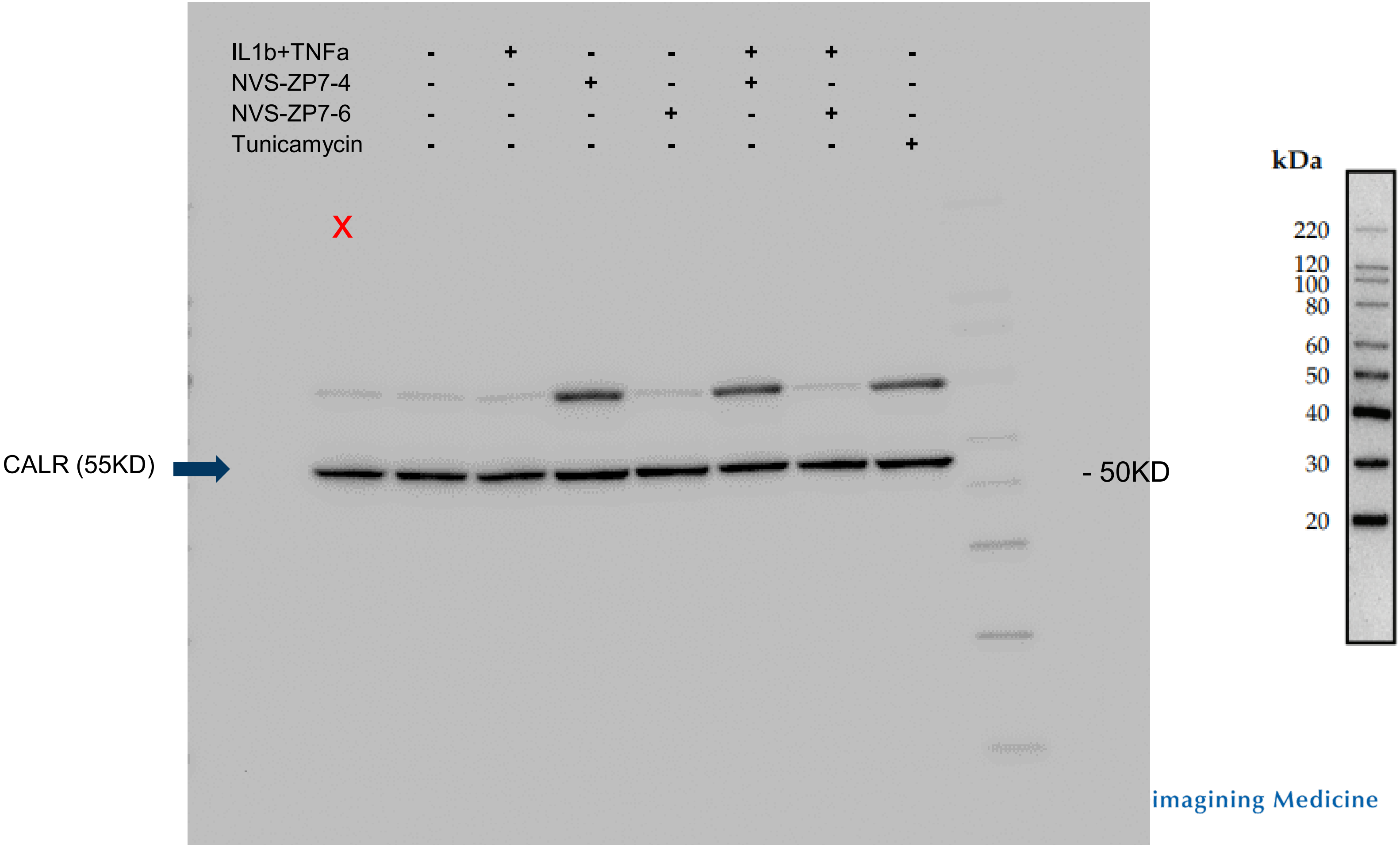

imagining Medicine

Fig 5A

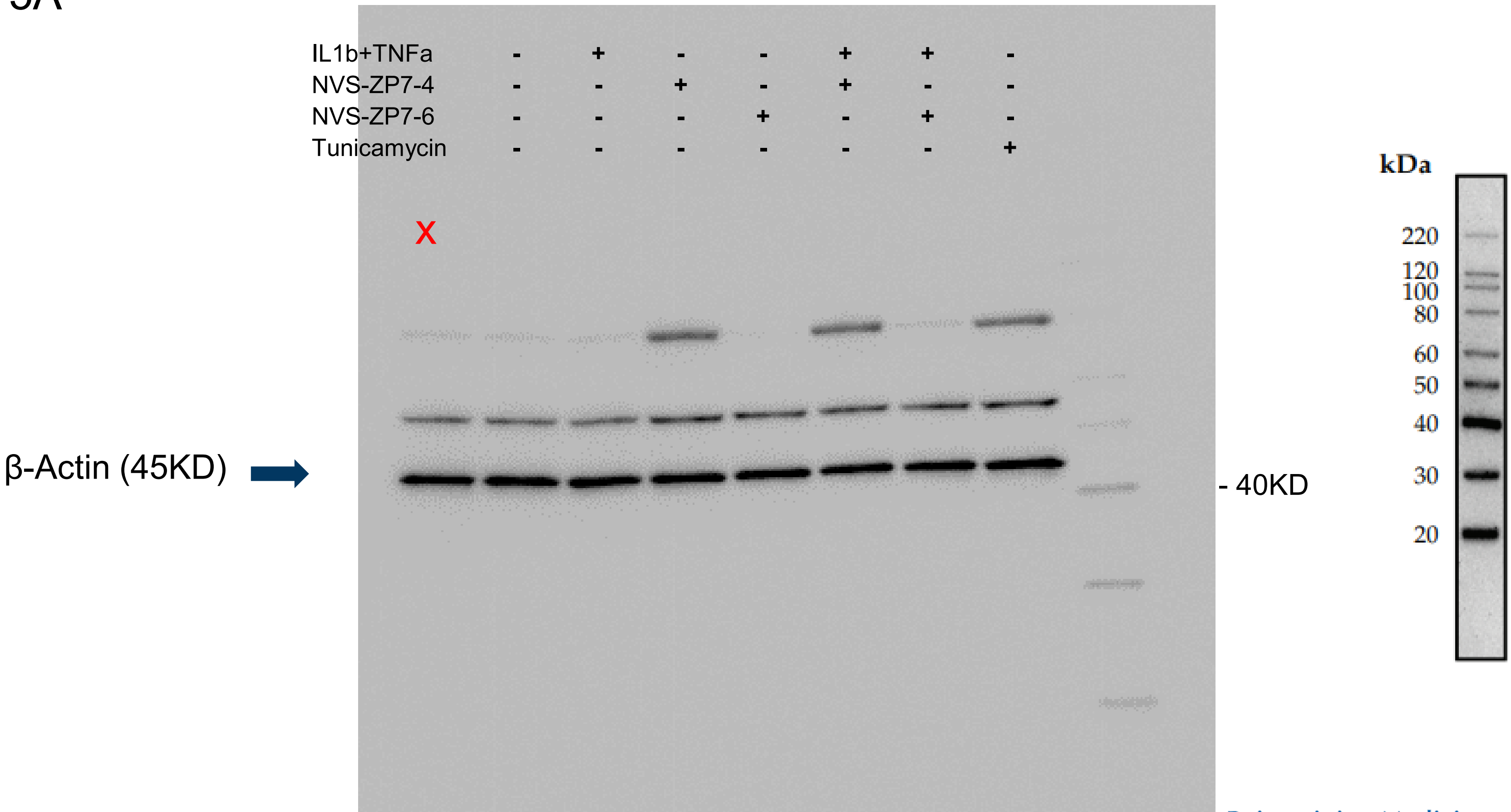
IL1b+TNFa - + - - + + -
NVS-ZP7-4 - - + - + - -
NVS-ZP7-6 - - - + - + -
Tunicamycin - - - - - - +
X
β-Actin (45KD)
- 40KD
kDa
220
120
100
80
60
50
40
30
20

Fig S5

IL1β/TNFα	-	+	-	+	-	-	+	+
NVS-ZP7-4	-	-	+	+	-	+	-	+
Zn/Pyr	-	-	-	-	+	+	+	+

BiP (78KD) ➡

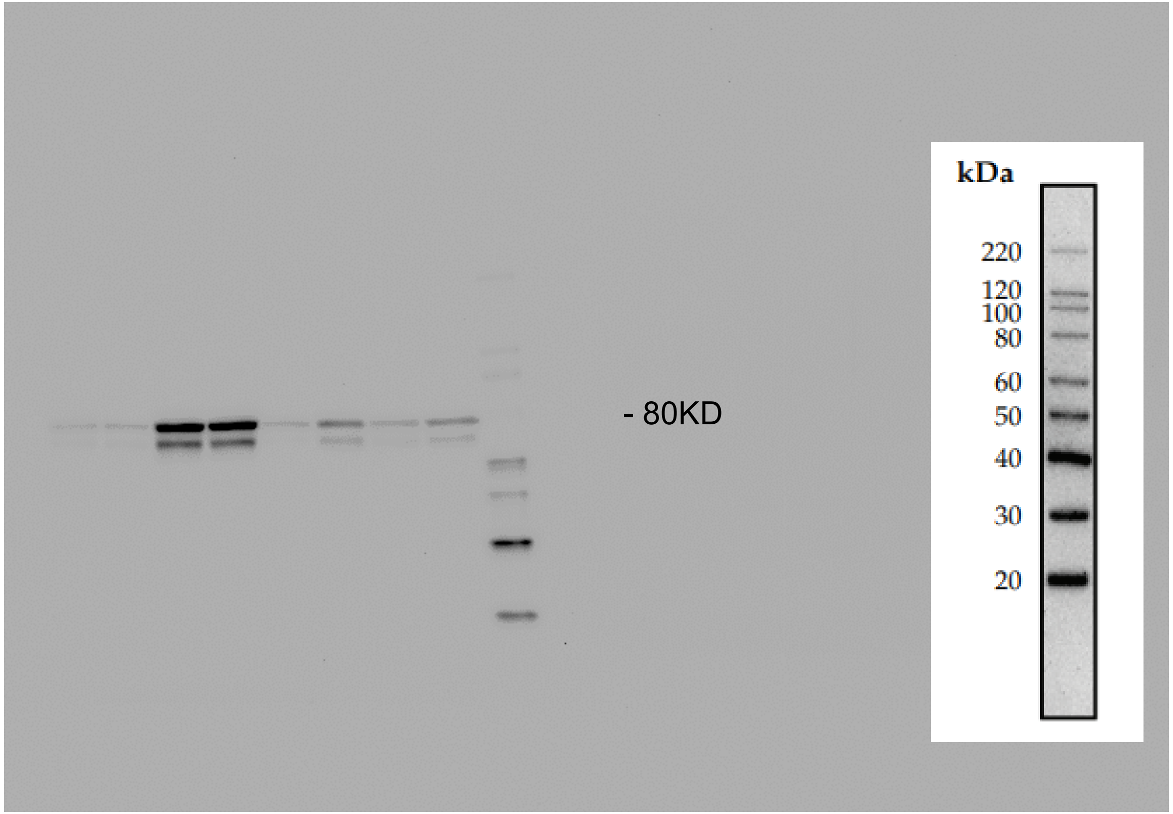

NOVARTIS | Reimagining Medicine

Fig S5

IL1β/TNFα	-	+	-	+	-	-	+	+
NVS-ZP7-4	-	-	+	+	-	+	-	+
Zn/Pyr	-	-	-	-	+	+	+	+

CHOP (29KD)

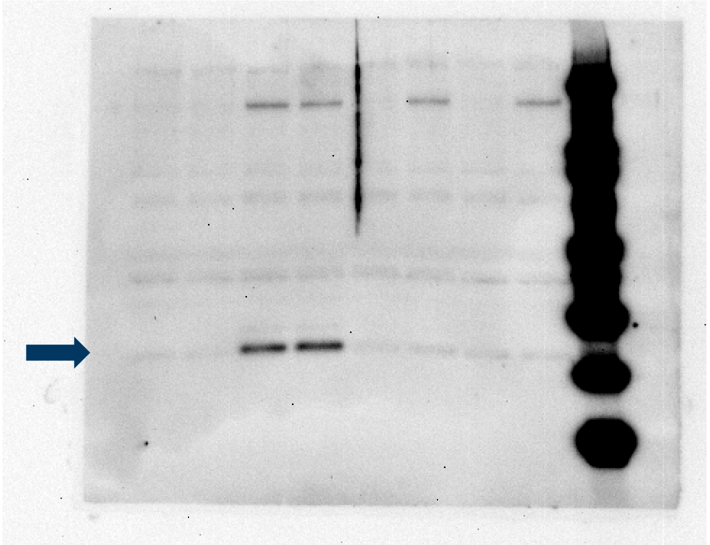

- 30KD

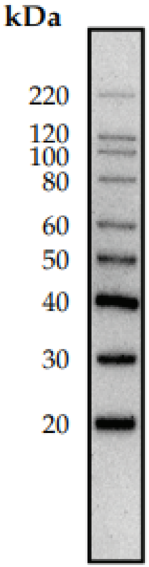

NOVARTIS | Reimagining Medicine

Fig S5

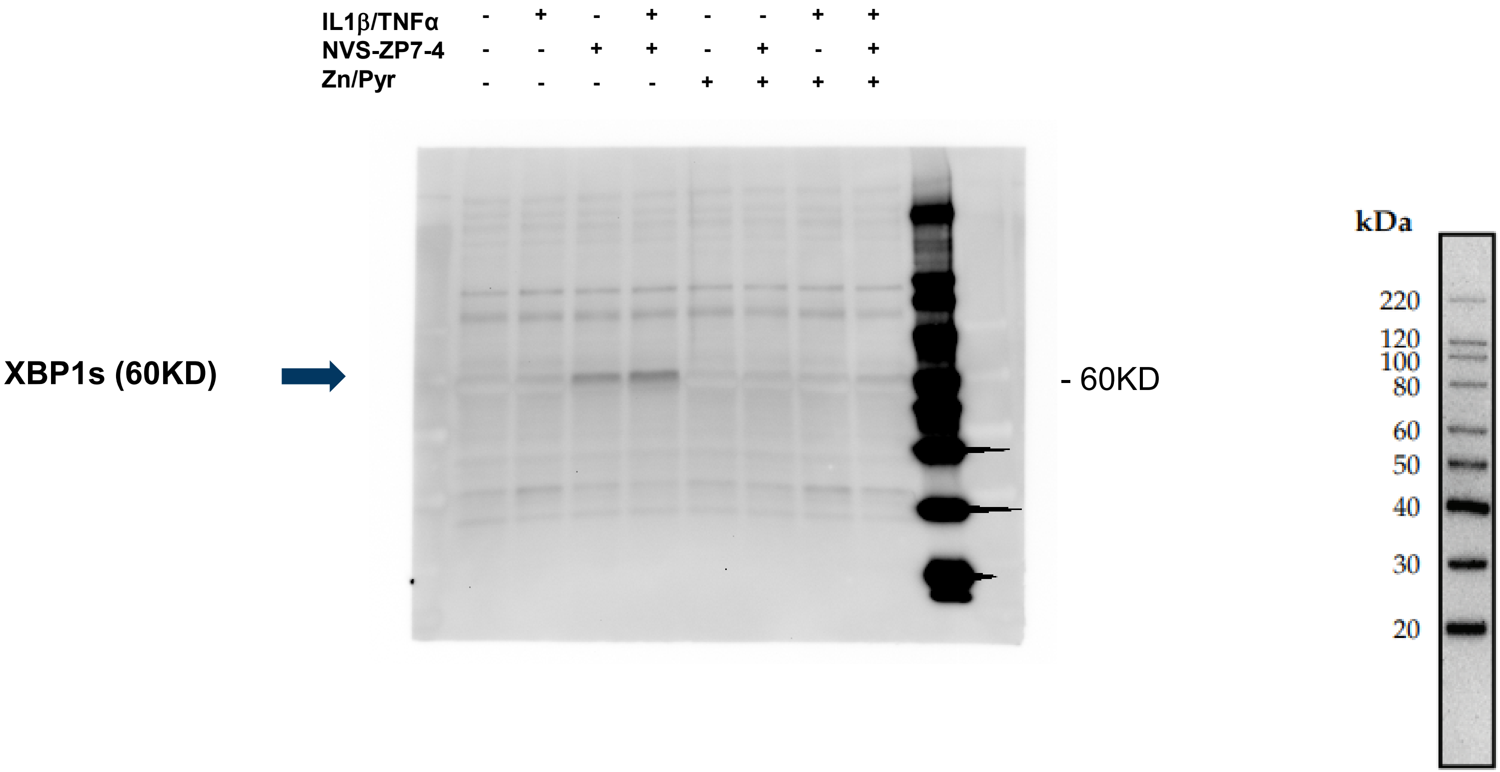

NOVARTIS | Reimagining Medicine

Fig S5

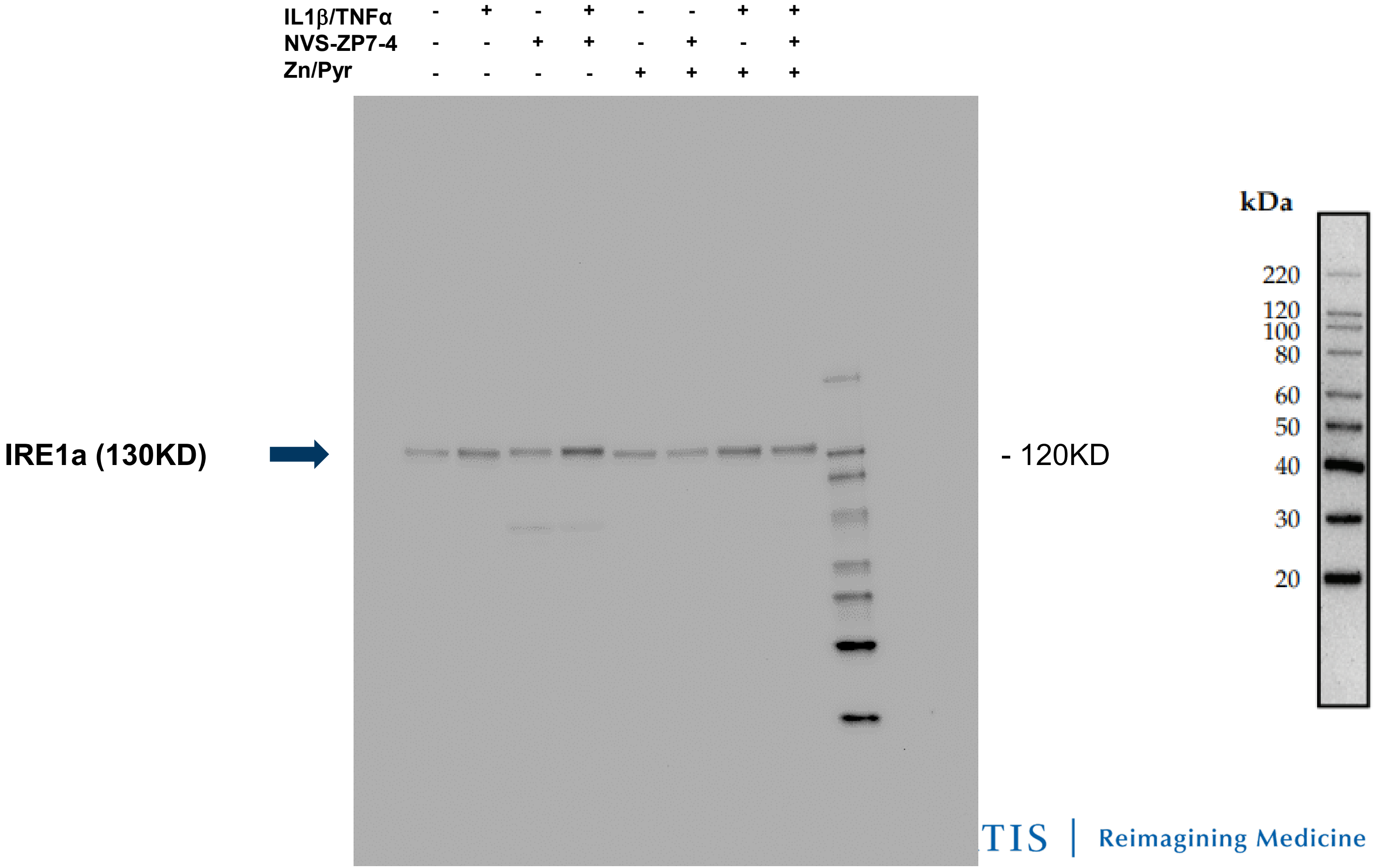

TIS | Reimagining Medicine

Fig S5

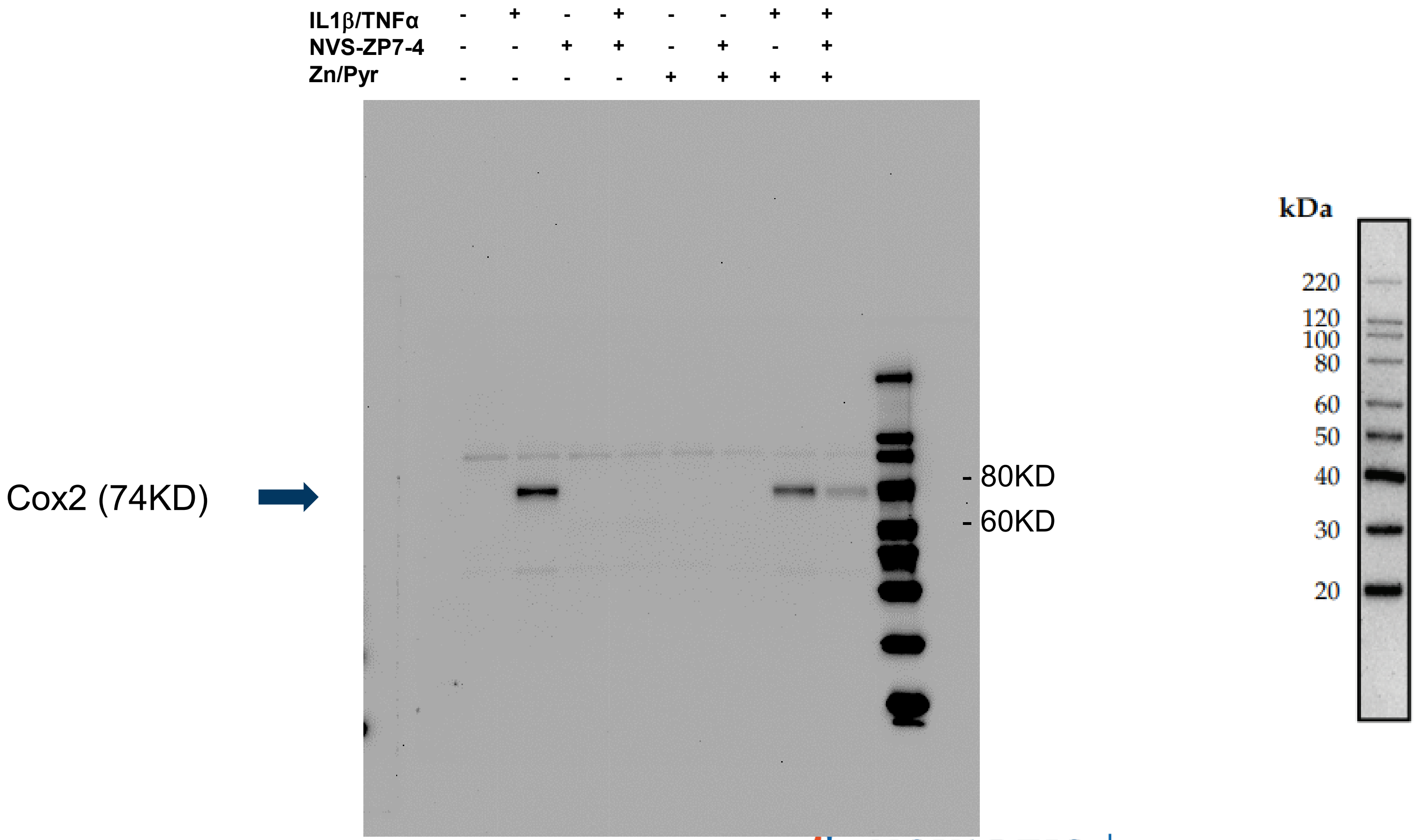

NOVARTIS | Reimagining Medicine

Fig S5

IL1β/TNFα	-	+	-	+	-	-	+	+
NVS-ZP7-4	-	-	+	+	-	+	-	+
Zn/Pyr	-	-	-	-	+	+	+	+

Occludin (65KD) →

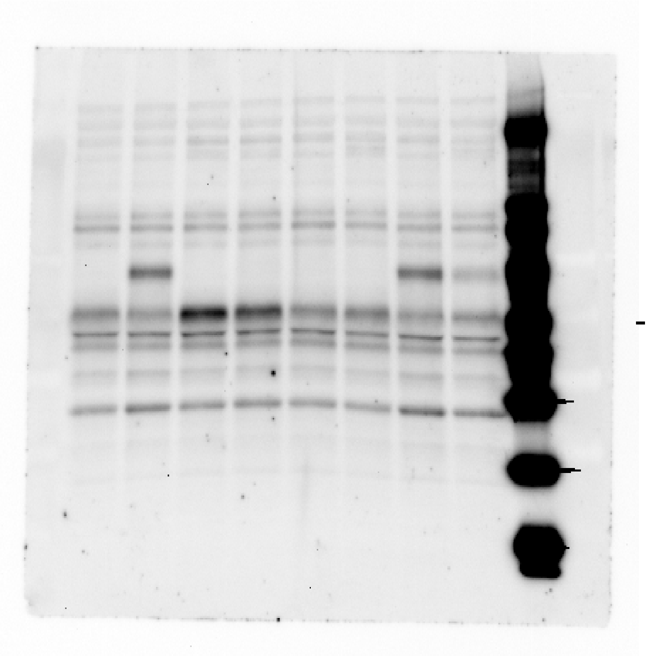

- 60KD

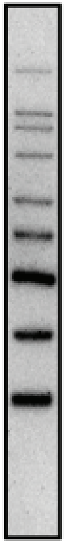

NOVARTIS | Reimagining Medicine

Fig S5

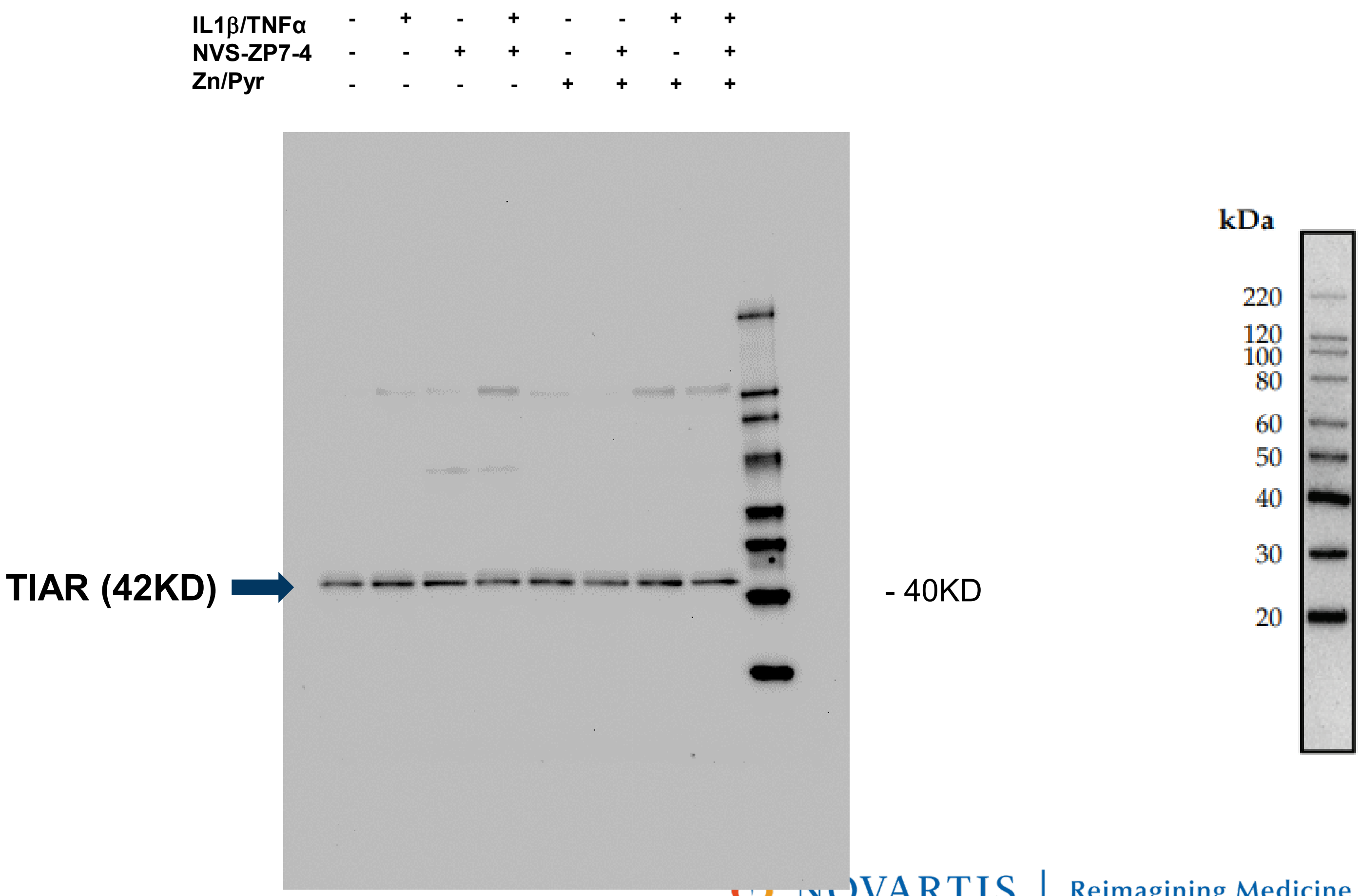

NOVARTIS | Reimagining Medicine

Fig S5

IL1β/TNFα	-	+	-	+	-	-	+	+
NVS-ZP7-4	-	-	+	+	-	+	-	+
Zn/Pyr	-	-	-	-	+	+	+	+

TIA1 (42, 44KD)

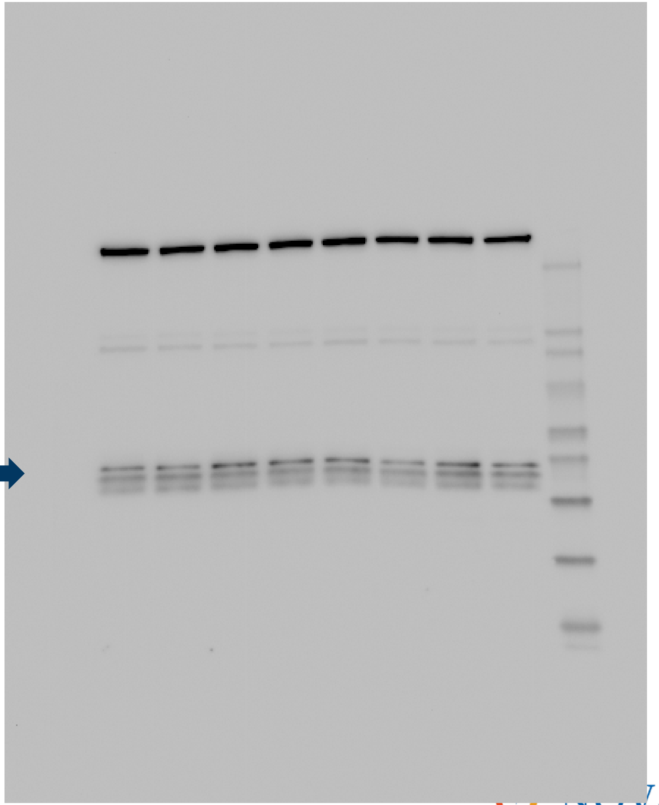

- 40KD

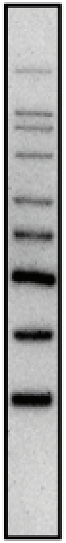

NOVARTIS | Reimagining Medicine

Fig S5

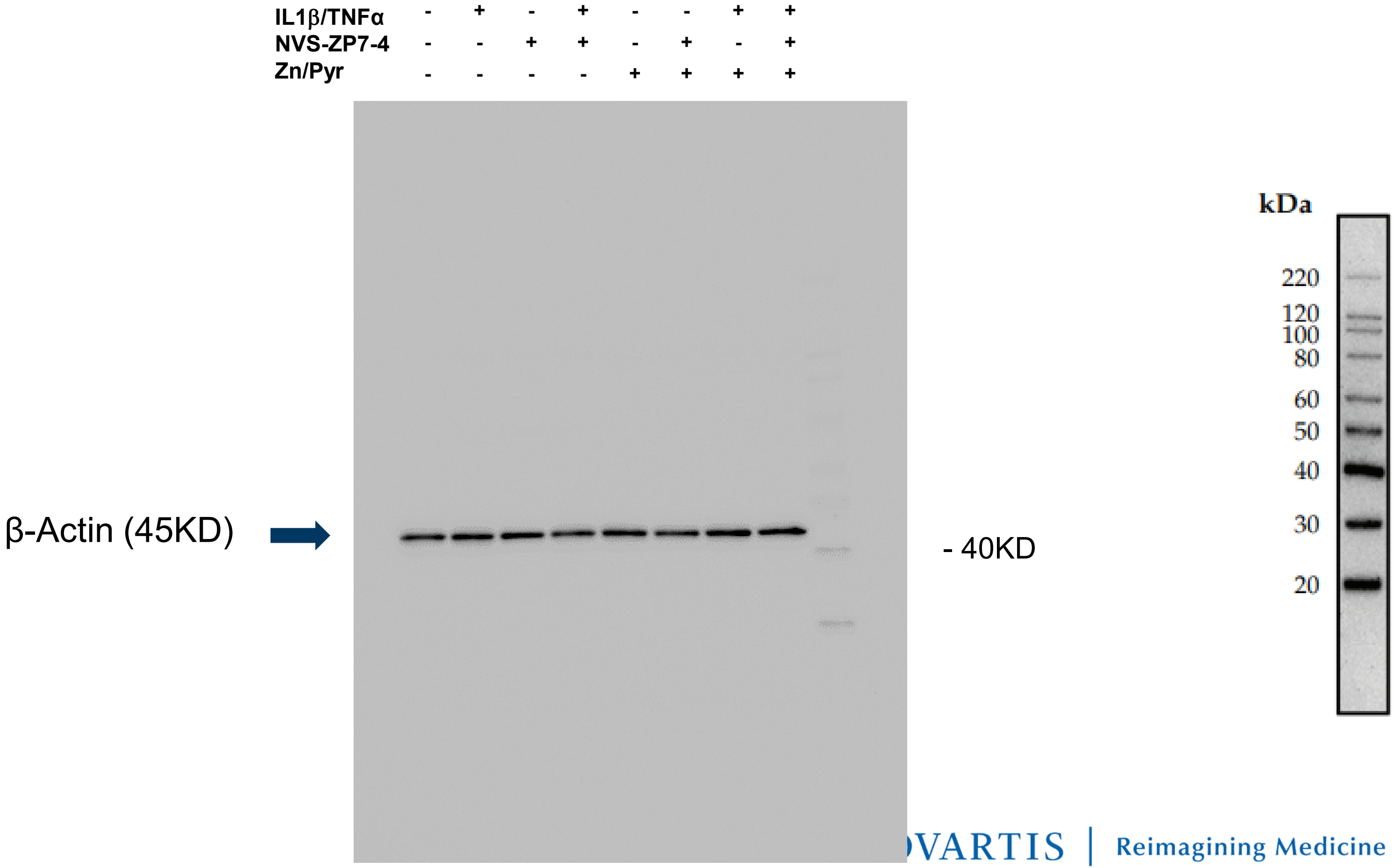
IL1β/TNFα - + - + - - + +
NVS-ZP7-4 - - + + - + - +
Zn/Pyr - - - - + + + +
β-Actin (45KD)
- 40KD
kDa
220
120
100
80
60
50
40
30
20
